# Experimental and computational advances on the study of Viscous Fingering: An umbrella review

**DOI:** 10.1016/j.heliyon.2021.e07614

**Published:** 2021-07-18

**Authors:** Andrés Pinilla, Miguel Asuaje, Nicolás Ratkovich

**Affiliations:** a Department of Chemical Engineering, University of Los Andes, Bogotá, Colombia; b Frontera Energy, Bogotá, Colombia

**Keywords:** Viscous Fingering, Miscible and immiscible displacements, Experimental and computational studies

## Abstract

During the production of heavy oil reservoirs, the movement of the fluids, namely oil and water, significantly affects the production rates. This movement is influenced by the mobility ratio and directly affects variables such as The *Water-Oil-Ratio (WOR)*, production costs, and recovery factor (RF). Moreover, Viscous Fingering, a phenomenon that describes the fluid movement through porous media, has been identified as the root cause of high-water production rates. Studying and comprehending this phenomenon is necessary to understand Oil & Gas companies' challenges nowadays to produce heavy oil. For example, this phenomenon has a direct impact on the assets managed by Enhanced Oil Recovery Techniques (EOR) that involves the injection of fluids such as polymer, water, and CO_2_ flooding, SAGD, VAPEX, CSP and ECSP, among others. Due to its importance, this paper review and highlights the main computational and experimental studies for over more than 30 years (from the late 1980s) about Viscous Fingering, especially in the oil industry. Also, the need for further studies involving the newest experimental and computational technologies and new novel methodologies for the comprehension of Viscous Fingering is discussed. This review aims to give an overview of the technological developments in the study of Viscous Fingering, not only to understand it but also to illustrate how scientists have been developing new technologies to overcome the consequences caused by this phenomenon.

## 1 Introduction

Before the main reading about the Viscous Fingering (*VF*) advancements of the last few decades, this section will briefly present some basic relevant concepts to understand this phenomenon. These basic concepts, definitions, and equations will contextualize the unfamiliar reader to multiphase-fluid flow through porous media, which describes the physics behind *VF*.

### 1.1 Rock definitions

Rock or porous material properties refer to the media's physical characteristics that affect the porous fluid flow.

- **Porosity** (∅): A porous media consists of a material capable of storing fluids inside geometrical structures called pores. The porosity measures the ratio of the volume occupied by the fluid and the total volume of the porous material. It is given by Eq. (1) where *V*_*f*_ is the volume occupied by the fluid and *V* is the total solid volume.

Eq. 1 $$\varphi =\frac{V_{f}}{V}$$

- **Absolute permeability** (***k***): Is a porous surface property that measures the ability to allow the flow of fluids in porous media.
- **Homogeneity and heterogeneity:** It refers to the distribution of porosity and permeability in a porous region. If it is homogeneous, it means that these properties are the same in the whole volume. On the contrary, if it is heterogeneous, they vary.

### 1.2 Fluid and multiphase flow definitions

- **Multiphase flows:** These are flows characterized by involving two or more fluids phases. These can be, for example, liquid-liquid such as the flow of oil and water; liquid-gas, i.e., the flow of oil and gas, among others. These flows are more complex than single-phase flows, as the transfer of momentum, mass, or energy can occur between the phases.
- **Density** (*ρ*): Refers to the mass per unit volume of a fluid.
- **Dynamic viscosity** (***μ***): Is a measure of the resistance of a fluid to flow. In multiphase flows in porous media, the highest viscosity contrast between the phases means more severe fingering.
- **Effective permeability** *(****k***_***i***_*)* **and relative permeability** *(****k***_***r***_*)*: In multiphase flows in porous media, the ability of the phase *i* to flow will be affected by the presence of the other phase in terms of volume fraction or saturation. Therefore, effective permeability refers to the ability of the phase *i* to flow in the presence of other immiscible fluids. On the other hand, relative permeability is the ratio between the effective permeability of the phase *i* at a certain volume fraction to the absolute permeability; it is explained in Eq. (2).

Eq. 2 $$k_{r}=\frac{k_{i}}{k}$$

- **Mobility and mobility ratio** *(****M****)*: Mobility is the ratio between the effective permeability of a phase and its dynamic viscosity; it is shown in Eq. (3). While the mobility ratio refers to the ratio between the mobility of the displacing fluid and the displaced fluid, as shown in Eq. (4).

Eq. 3 $$Mobility=\frac{k_{i}}{\mu_{i}}=\frac{kk_{r}}{\mu_{i}}$$

Eq. 4 M=MDisplacing@ResidualoilvolumefractionMDisplaced@Connatewatervolumefraction

where *k*_*i*_ refers to the effective permeability of the phase *i* and *k* refers to the absolute permeability. Also, *M*_*Displacing*_@*Residual oil volume fraction* and *M*_*Displaced*_*@Connate water volume fraction* are referred to the mobility of the phases at irreducible saturations. These are usually determined with experimental measurements.

- **Capillary pressure** *(****P***_***c***_*)*: For immiscible multiphase flows through porous media, the capillary pressure results from the forces between the immiscible fluids and the pore walls. It allows determining how easy fluids transports through the porous media. It is shown in Eq. (5).

Eq. 5 $$P_{c}=P_{non-wetting\;phase}-P_{wetting\;phase}$$

- **Sweep efficiency** refers to the fraction of oil displaced from the porous media by displacing fluid.
- **Miscibility:** It refers if two or more fluids are capable of mixing in a homogeneous solution. If the fluids mix themselves, they are referred to as a miscible solution. On the contrary, if the phases do not mix themselves, they are an immiscible solution, separated with a visible interface.
- **Interfacial tension:** When two fluids are immiscible, they are separated by interface or “film,” which defines the transition between the two immiscible phases. Interfacial tension is referred to the attraction forces between the molecules of the phases at the interface.
- **Displacement stability:** The displacement refers to move one fluid with other; for example, move oil with water. The displacement front is the interface between the displacing fluids. When this front is sharp like a “film,” it is a stable displacement called a piston-like displacement. When the displacement front is not sharp, instead has *VF* patterns, it is an unstable displacement.
- **Molecular diffusion:** Occurs in miscible *VF;* it refers to the mass transport of the phases due to a concentration gradient.
- **Mechanical dispersion:** Occurs in miscible *VF* when the phases are mixed due to external forces, such as a pressure gradient or other induced velocity variations that favor advective (or convective accounting diffusion in the same process) transport.

### 1.3 Experimental devices

**Figure 1:**
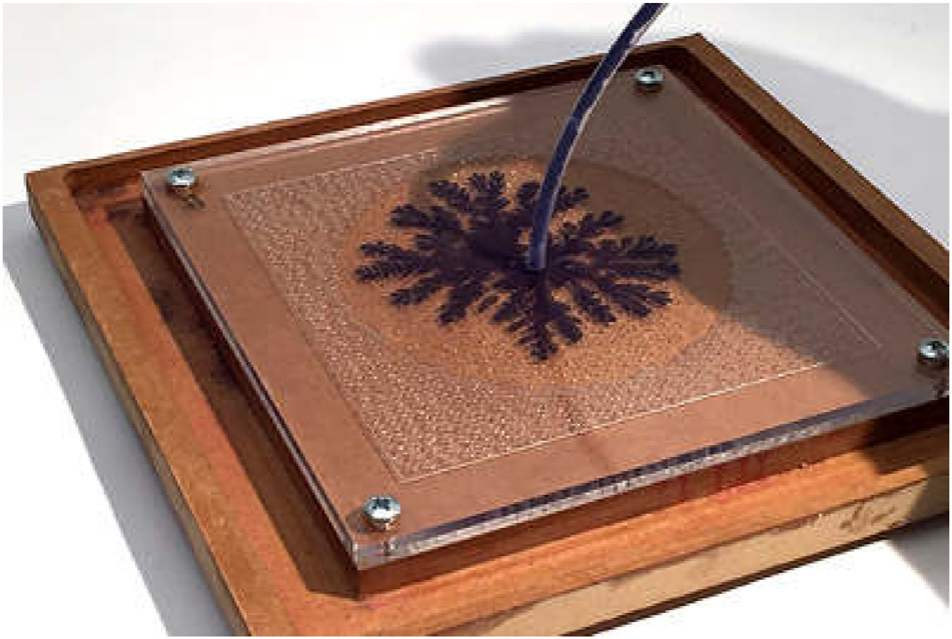
Hele-Shaw experimental setup performing a Viscous Fingering experiment. Adapted from Salmaan Craig [1].
- **Micromodels:** *2D* setups that resemble a porous media by imprinting porous patterns in between two flat plates. These patterns can be homogeneous or heterogeneous, even resembling authentic porous patterns found in rock samples. Some examples are shown in Figures 7 and 8.
- **Sand packs:** *2D* and *3D* experimental setups larger than the Hele-Shaw cells and the micromodels. It is made of packed gravel, sand, or other materials.
- **Cores:** *3D* rock samples of cylindrical shape extracted directly from the reservoirs.

- **Hele-Shaw cell:** *2D* setups that consist of displacing fluids in between two flat plates. In these cases, the porosity is equal to one, and the permeability is infinite. An example of these experimental setups is presented in Figure 1.

## 2 General framework: flow through porous media and the Saffman-Taylor instability

**Figure 2:**
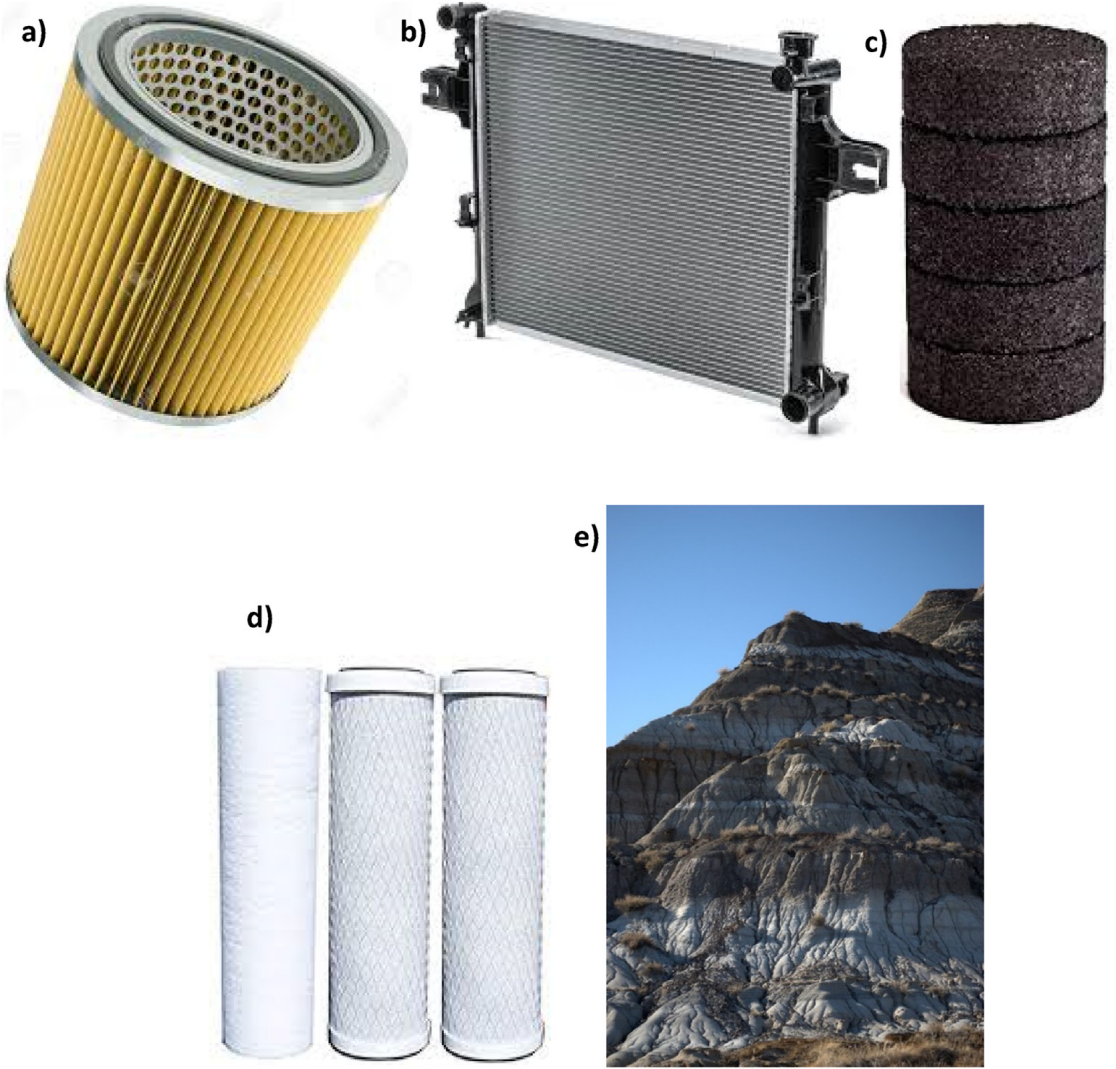
Some examples of daily applications of porous media: a) Air filters for combustion engines, b) Radiators for heat exchange, c) Activated carbon filters for water treatment, d) Reverse osmosis filters for water desalinization, and e) a mountain, where the horizontal layers can show different porous properties.

The flow-through porous media has been studied from a long time ago, beginning in the 19^th^ century, where Henry Darcy [2] developed a constitutive model based on his water flow studies through packed sand. Flow-through porous media is present in nature, and it appears in several modern and industrial applications. For example, in the flow-through packed beds or separation units in the chemical and food industry. In civil engineering for the flow-through sanitation systems, electrical engineering, and physics for the flow-through power cells. Moreover, this flow has a significant impact in the energy sector: geology, petroleum engineering, and the O&G industry, where energy in the form of oil and gas is stored in porous rocks. To illustrate the importance of flows through porous media, Figure 2 are presented some modern everyday applications.

The first studies of flows through porous media were made to understand the flow hydrodynamics through these systems, exclusively for single-phase flows. Studies like those conducted by Henry Darcy [2], Jules Dupuit [3], and Philipp Forchheimer [4], around the 19^th^ century, allowed to propose mathematical expressions that described these kinds of flows and nowadays known as constitutive laws that describe the flow through porous media and which are widely used for several applications—mainly by the prediction of the pressure drop by a velocity field, affected by the viscous and inertial resistance which are function or dependent of the porous material and the fluids involved.

However, the experimental study of the fluid dynamics in porous materials can be complex since they are usually dark and opaque, with obvious optical limitations. To overcome this issue, Henry Hele-Shaw [5] developed a simple experimental setup that consisted of two parallel transparent plates where fluids could be visualized called the Hele-Shaw cell, previously presented in Figure 1. Moreover, the fluid dynamics of this setup were equal to those previously proposed by Darcy or Dupuit, and Forchheimer. The Hele-Shaw cell was developed in the late *1800's*, and it has been widely used to study micro-fluidics.

**Figure 3:**
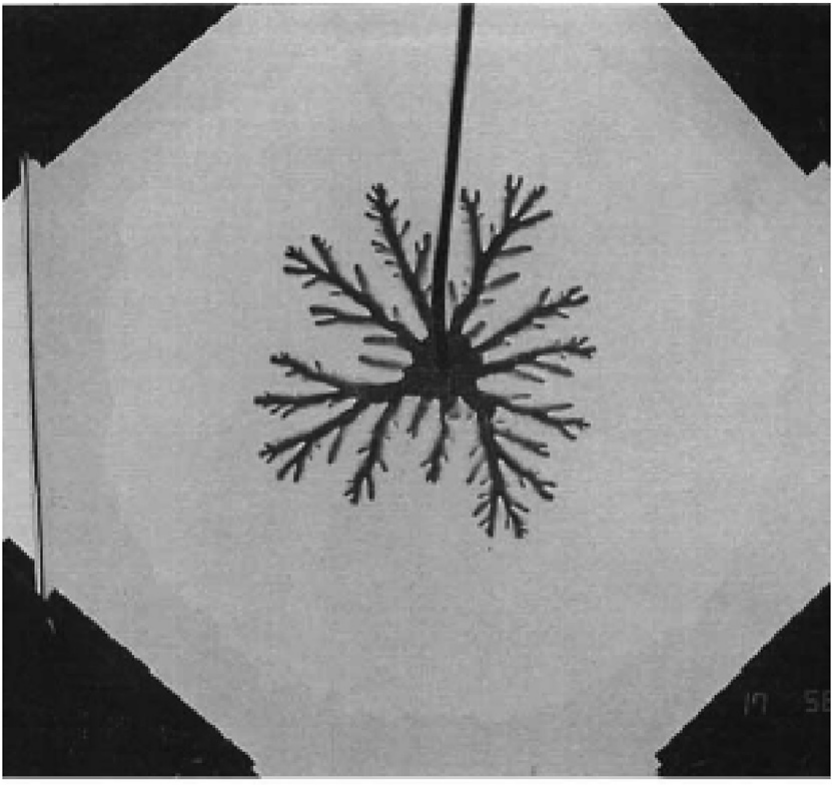
Experimental visualization of miscible VF with a Hele-Shaw setup under a radial displacement using water and glycerin. Image adapted from Chen [9].

Moreover, 50 years after its invention, it was used to study multiphase flows, as mentioned by Homsy in his classical review [6]. These very first studies were made by Saffman and Taylor [7] and Chuoke [8], who reported the appearance of unseen flow patterns, which they called Viscous Fingering (VF) due to the “*finger”* structure they tend to generate on the displacement front. Also, the VF was early characterized for presenting interaction dynamics between them as they tend to collide and coalesce, split, birth along the displacement front, and flow in several directions. These new flow patterns are presented in Figure 3. VF is a phenomenon that describes miscible and immiscible multiphase flows through porous media, and it is mainly caused by the difference in the viscosity ratio of the involved phases. For example, during a two-phase displacement, the phase with the lowest viscosity will move or displace faster than its more viscous counterpart, causing interfacial instabilities that will lead to the appearance of finger-shaped patterns, as shown in Figure 2. Hill [9] observed this VF phenomenon earlier in an industrial application, before Saffman, Taylor, and Chouke. He reported VF on packed columns for vertical downward flows during sugar refining operations involving the displacement of sugar liquors with water.

These earlier studies of VF were focused on explaining the instabilities between the interface of two fluids and the development of mathematical expressions that could describe the displacement of both fluids in porous media. Saffman, Taylor, and Chouke developed the first theories and mathematical expressions to explain and describe VF. Chouke [8] developed a theory based on analyzing the linear displacement of two immiscible fluids in a Hele-Shaw system. As a result, a one-dimensional model was created and validated against experimental data. On the other hand, Saffman and Taylor [7] developed a theoretical analysis focus on the evolution and growth of a single finger, which in their study was referred to as the penetration of a fluid into a more viscous one, obtaining a one-dimensional mathematical expression to describe the growth of VF.

From these first studies, it was also remarkable that the authors recognized the direct link between this phenomenon and oil reservoirs. Before VF's discovery, geologists and oil engineers knew that water moved faster than oil from an interfacial instability causing channelings, which are fingers. At that time, the displacement theory was based on the Muskat [10, 11], and Buckley-Leverett [12] models, which did not account for VF as it was unknown. On the one hand, the Muskat model was developed by Morris Muskat in the mid 1930's and it assumed that the displacement of oil by water was piston-like, therefore, assuming a sharp interface between them. On the other, the Buckley-Leverett model was developed by E. Buckley and M. C. Leverett in the mid 1940's which was not based on a sharp displacement front. On the contrary, it recognized that the displacement front could be intermingling on an advancing interfacial region.

Since the discovery of VF, the O&G industry has been very interested in the study and comprehension of VF as it was recognized as the root cause of many issues in the reservoir like unstable fluid displacements, the decadency on sweep efficiency, and the high water production rates, especially for heavy oil reservoirs where the viscosity ratio favors the occurrence of this phenomenon. As mentioned before, VF is stronger under large viscosity ratios, then it severely affects the production of heavy oil reservoirs, significantly influenced by strong aquifers and mature oil fields or under EOR techniques that involve the injection of fluids. Therefore, this phenomenon also plays an essential role for EOR's such as waterflooding, polymer and chemical flooding, *CO*_*2*_ flooding; and even thermal EOR's such as VAPEX and SAGD, among others. From decades of study, some reviews have been made about the comprehension of this phenomenon. Two examples are the reviews made by George M. Homsy [6] in the late 1980s and the one made by Saleh Tanveer [13] in the late 1990's. Moreover, these reviews manly treated the advancements on fluid mechanics about this phenomenon leaving aside experimental and computational contributions on this field.

**Figure 4:**
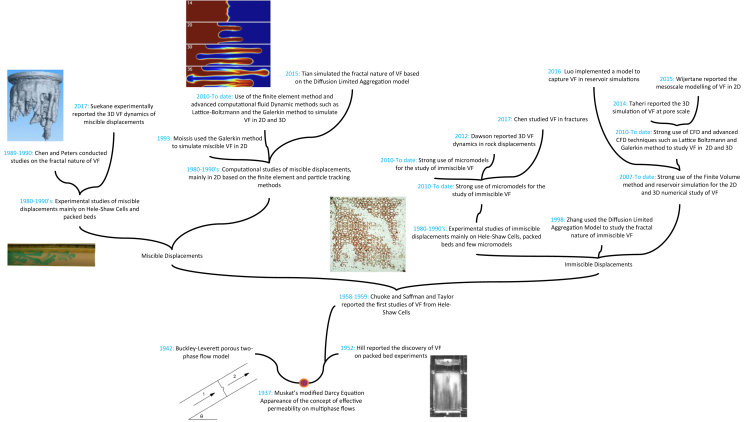
Summary of the history behind the study and comprehension of VF.

In this context, this article will review all the experimental and computational studies carried on after the late 1990's up to this date. The reader should refer to Homsy [6], and Tanveer [13] works to understand VF further, as they mainly treat the fluid dynamics of miscible and immiscible flows through porous media. Overall, this article includes the advancements in experimental techniques that have helped to understand further the flow dynamics of this phenomenon and the contributions of the computational engineering that have helped in modeling VF on several length scales supporting the experimental studies and going even beyond them. To contextualize the reader, Figure 4 briefly presents a diagram that summarizes the significant advancements and discoveries related to the study of VF, going from the distinction of miscible and immiscible VF to the latest contributions on the *3D* study of this phenomenon.

**Figure 5:**
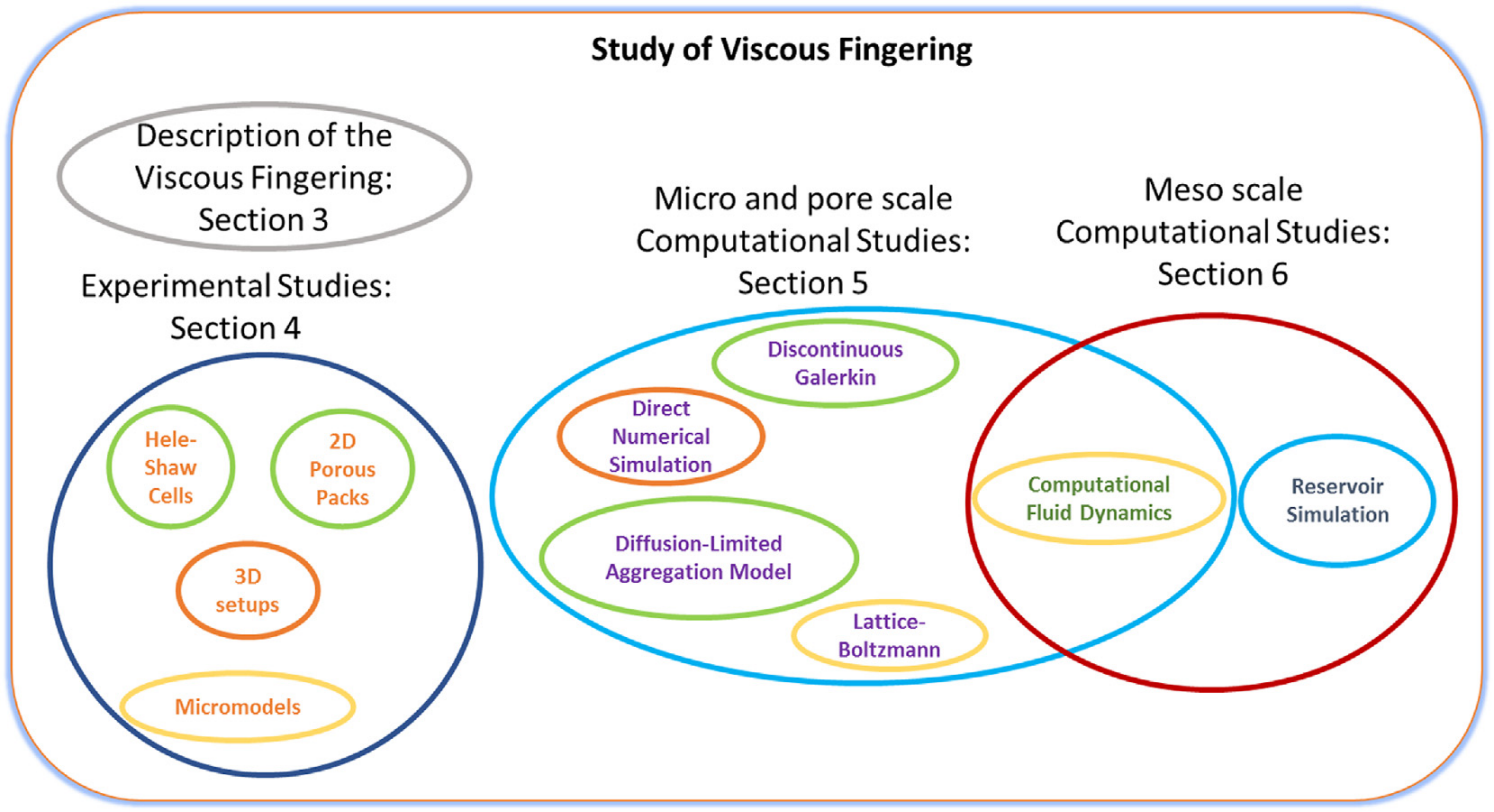
Illustrative division of the reviewed topics on the study of VF.

This review, which was based on more than 100 articles, has been organized in the following sections. Section 3 will be briefly reviewed some of the main features of VF. Again, the reader should refer to Homsy [6] and Tanveer [13] to provide complete insight into VF theory and flow dynamics. Section 3 will focus on the experimental advancements of miscible and immiscible displacements at multiscale. Section 4 will present the computational advancements made so far about the microscale modeling of VF for miscible and immiscible displacements. Finally, Section 5 will review the computational advancements in modeling this phenomenon at mesoscale and Computational Fluid Dynamics (CFD). Figure 5 shows a diagram that will help understand the division of this review.

## 3 Basic description of the VF dynamics

**Figure 6:**
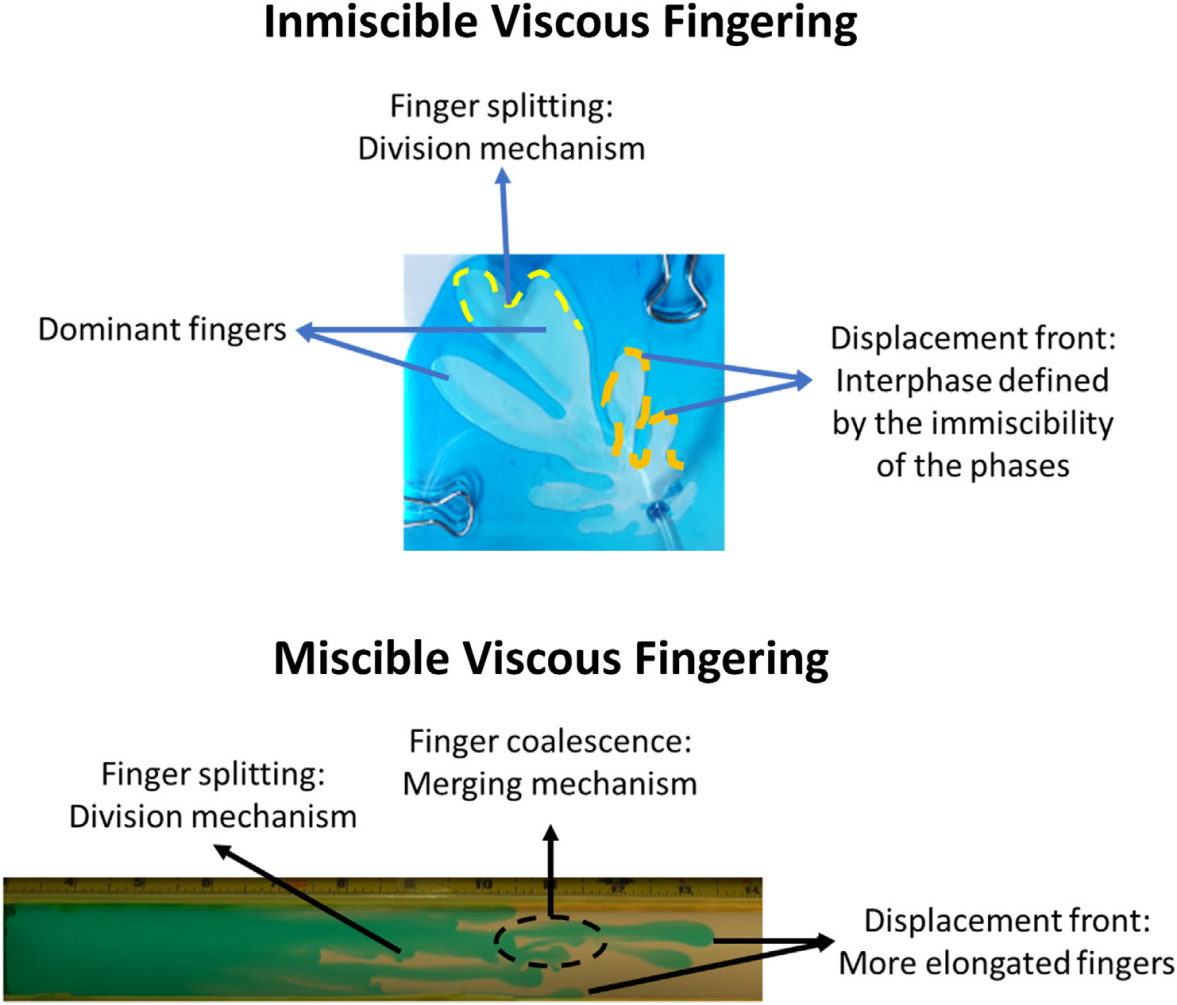
Visual differences and features of Immiscible radial Viscous Fingering and Linear Miscible linear Viscous Fingering. Miscible displacement image adapted from Malhotra [14]. Immiscible fingering is characterized by having thicker fingers, while miscible fingers are thinner and elongated. Also, linear displacements refer to displacements in a single vertical or horizontal direction. On the contrary, radial displacements are referred to as those flowing radially.

As it was previously mentioned, VF is the characteristic flow pattern in multiphase flows in porous media. However, according to the miscibility of the displacement, it can be divided into two kinds, immiscible and miscible displacements. The first ones, from now one referred to as IVF (Immiscible Viscous Fingering), are characterized for having an interface, where the main driven force that causes the *finger* patterns at the displacement front, or interface, is the viscous gradient caused by a difference on the viscosity of the phases. On the other hand, the miscible displacements, from now on referred to as MVF (Miscible Viscous Fingering), are characterized for not having an interface between the displacing phases and having strong diffusive effects caused by variations in concentration. Therefore, miscible fingering is a problem governed by two phenomena: i) mobility gradient or differences between viscous forces, and ii) diffusive effects or gradients of concentrations. An illustrative description of these two kinds of displacements is presented in Figure 6.

Despite Figure 6 shows some basic features of the IVF and MVF flow patterns, extensive studies have been conducted to propose mathematical models capable of describing both flow dynamics. For example, the works of Homsy [6] and Tanveer [13] have summarized the leading theory behind both displacements, going from the Hele-Shaw flow to approaches that describe the flow through porous media. Nevertheless, various researchers have conducted further studies of this phenomenon proposing new concepts and models that accurately describe its fluid dynamics.

Section 4 will discuss the experimental studies addressed to comprehend the VF phenomenon fully in the following section. Ranging from the usage of several fluids with a wide range of physical and rheological properties, to the usage of experimental devices that have allowed its study from the *2D* microscale to even the *3D* macroscale, and its incidence on essential fields such as oil recovery and *CO*_*2*_ sequestration. These experimental studies have allowed us to comprehend the fractal nature of VF displacements, the influence of the porous media and fluid properties on its flow dynamics, and the influence of external sources to change its dynamics, such as the usage of heat transfer and chemical reactions, for example in the study of Enhanced Oil Recovery (EOR) for the oil industry. It is essential to highlight that this review does not include the effect of chemical reactions; again, the reader should refer to the comprehensive review of De Wit about this exciting topic [15]. Finally, Section 5 will discuss the modelling of this phenomenon, most of the cases derived from mathematical models obtained from these experimental observations, to predict its behavior for practical purposes. These studies vary from the proposal of *2D* models to the *2D* and *3D* modelling based on reservoir simulator codes based on the diffusivity equation to the modelling based on the governing equations of fluid flow.

## 4 Experimental studies of VF: Hele-Shaw cells, micromodels, porous networks, and *3D* approaches

The study of miscible and immiscible displacements through porous media can be made from a wide variety of experimental setups. The Hele-Shaw cells, porous micromodels, and packed setups allow easy visualization and measurement of *2D* linear and radial displacements, up to *3D* experimental setups where visualization and measurement techniques became very complicated and expensive. In this section are reviewed the experimental studies made in *2D* and *3D* about VF. Tables 1 and 2 are summarized the experimental setups and fluids used in the reviewed studies, along with the main physical properties for miscible and immiscible displacement studies, respectively^1^^,^^2^.

**Table 1:** Summary of the experimental setup and fluids used for miscible displacement studies.

Author	Experimental setup	Fluids	Density $\left(\frac{kg}{m^{3}}\right)$	Viscosity (*P a s*)	Porosity [0–1]	Permeability (*m*^2^)
Hu 1985 [16]	Packed porous media	- Water - Glycerin	NR	NR	0.38	7 × 10^−11^
Chen 1987 [17]	Radial Porous Network	- Water - Glycerin	NR	0.001 1.2	-	-
Chen 1987 [18]	Radial Hele-Shaw Cell & Radial Porous Network	- Water - Glycerin	NR 1260	0.001 1.05	-	-
Chen 1989 [9]	Radial Hele-Shaw Cell	- Water - Glycerin	NR 1260	0.001 1.05	-	-
Peters 1990 [19]	Packed porous media	- Water - Glycerin	NR	NR	0.4	1.97 × 10^−11^
Pope 1996 [20]	Sandpack & Packed porous media	- KCl-water solution - Guar gum solutions at different concentrations	RTM	- -	NR	NR
Cuthiell 2006 [21]	Sandpacks	- 4 Oils - Toluene - n-butane	NR	*1st oil*: 4.56 *2nd oil*: 5.56 *3rd oil*: 6.63 *4th oil*: 49	NR	4 × 10^−10^ 4 × 10^−11^
Malhotra 2015 [14]	Linear Hele-Shaw cell	- Water - Glycerol solutions	NR	NR 10^−3^-1.125	-	-
Suekane 2017 [22]	Packed bed	- NaCl Solution - Glycerol solution 60% - Glycerol solution 85% - Glycerol - NaI Solution - NaI–NaCl solution 1 NaI–NaCl solution 2	1.07 × 103 1.15 × 103 1.22 × 103 1.26 × 103 1.07 × 103 1.15 × 103 1.22 × 103	1.19 × 10^−3^ 1.25 × 10^−3^ 8.27 × 10^−3^ 9.06 × 10^−1^ 9.16 × 10^−4^ 1.25 × 10^−3^ 1.74 × 10^−3^	0.51	1.3 × 10^−10^

**Table 2:** Summary of the experimental setup and fluids used for immiscible displacement studies.

Author	Experimental setup	Fluids	Density $\left(\frac{kg}{m^{3}}\right)$	Viscosity (*P a s*)	Interfacial tension (*N/m*)	Porosity	Permeability
Chen 1987 [17]	Radial Porous Network	- Oil - Water - Glycerin	NR	NR 0.001 1.2	Oil-Water: 0.0346 Oil-Glycerin: 0.0203	-	-
Chen 1987 [18]	Radial Hele-Shaw Cell & Radial Porous Network	- Oil - Glycerin	740 1260	0.001 1.05	0.02	-	-
Chen 1989 [9]	Radial Hele-Shaw Cell	- Oil - Glycerin	740 1260	0.001 1.05	0.02	-	-
Peters 1987 [23]	Sandpack	- Oil - Water	950 990	0.108 0.001	0.002	0.35	9.93 × 10^−12^
Lajeunesse 1999 [24]	Linear & Radial Hele-Shaw cell	- Air - 4 Oils	NR	NR *1st oil*: 0.0087 *2nd oil*: 0.0199 *3rd oil*: 0.1064 *4th oil*: 0.469	NR	-	-
Lindner 2002 [25]	Linear Hele-Shaw cell	- Air - Xanthan - Polyethylene Oxide (POE)	NR	NR	Air-Xanthane: 0.072 Air-POE: 0.063	-	-
Kawaguchi2003 [26]	Linear & Radial Hele-Shaw cell	- Air - HPMC-silica solution - PPG-silica suspension	RTM	NR	RTM	-	-
Kawaguchi 2004 [27]	Radial Hele-Shaw cell	- Water - Emulsion of Silicone oil and an aqueous solution of HPMC - Aqueous solution of HPMC	RTM	NR	NR	-	-
Li 2009 [28]	Radial Hele-Shaw cell	- Air - Castor oil	NR 1	NR	0.048	-	-
Sinha 2009 [29]	Radial Hele-Shaw cell	- Air - Castor oil - Olive oil	NR	NR	NR	-	-
Jamaloei 2010 [30]	Micromodel	- Crude oil - Solution of surfactant, ethanol, and xanthan	927.4 NR	0.0057	6.5 × 10^−5^	0.358	1.79 × 10^−12^
Jamaloei 2016 [31]	Micromodel	- Water - Crude oil	NR	NR 0.0806	0.0084	0.358	1.79 × 10^−12^
Jamaloei 2016 [32]	Micromodel	- Crude oil - Solution of surfactant, ethanol and calcium, magnesium, and sodium chloride	927.4 NR	0.0806 9 × 10^−5^	0.0084	0.35	2.35 × 10^−12^
Pei 2011 [33]	Micromodel	- Crude oil - Brine - NaCl solutions with sodium carbonate - NaCl solutions with sodium hydroxide	930.2 NR NR NR	0.325 NR NR NR	RTM	NR	NR
Pei 2012 [34]	Sandpack Micromodel	- Crude oil - Brine - NaOH solutions with surfactant SLPS - NaOH solutions with surfactant ORS	947.2 NR NR NR	2 NR NR NR	RTM	42.19–54.1 (RTM)	1.938 × 10^−12^-2.23 × 10^−12^ (RTM)
Doorwar 2011 [35]	Micromodel	- 3 Oils - Brine - HPAM solution - Alkaline surfactant - Glycerol solution	NR	*1st oil:* 1 *2nd oil*: 10 *3rd oil*: 0.006 *HPAM solution*: 0.1 *Glycerol solution*: 0.006	NR	NR	NR
Doorwar 2015 [36], 2017 [37]	Micromodel Cores	- Synthetic and crude oils - 2 Brines	NR	RTM	NR	*Core*: 0.29	*Core*: 5.92 × 10^−12^
Zhang 2011 [38]	Micromodel	- Water - Polyethylene glycol 200 (PEG200) - Hexane (HA) - Dodecane (DD) - Hexadecane (HD) - Mineral oil (MO)	NR	2.92 × 10^−3^ 0.33 × 10^−3^ 1.35 × 10^−3^ 3.34 × 10^−3^ 77.6 × 10^−3^	PEG200-HA: 12.35 × 10^−3^ PEG200-DD: 13.85 × 10^−3^ PEG200-HD: 14.27 × 10^−3^ Water-HA: 49.71 × 10^−3^ Water-DD: 51.21 × 10^−3^ Water-HD: 52 × 10^−3^ Water-MO: 36.32 × 10^−3^	0.39	NR
Zhang 2011 [39]	Micromodel	- Water - *CO*_*2*_	998 825	7.6 × 10^−5^ 9.82 × 10^−4^	0.0287	0.39	RTM
Wang 2013 [40]	Micromodel	- Water - *CO*_*2*_	990 490	3.6 × 10^−5^ 6.4 × 10^−4^	0.0262	0.4	NR
Sharma 2012 [41]	Micromodel	- Water - 3 Crude oils	NR	NR *1st oil: 8* × 10^−3^ *2nd oil: 9* × 10^−3^ *3rd oil*: 0.4	NR	0.47	9.86 × 10^−13^
Zhao 2019 [42]	Cores Micromodel	- Different wetting fluids - Different non-wetting fluids	NR	RTM	NR	*1st core*: 0.22 *2nd core*: 0.18 *3rd core*: 0.27 *4rd core*: 0.28 *Micromodel*: RTM	*1st core*: 4.73 × 10^−13^ *2nd core*: 1.57 × 10^−13^ *3rd core*: 2.17 × 10^−14^ *4rd core*: 2.17 × 10^−14^ *Micromodel*: 1.283 × 10^−12^
Chen 2017 [43]	Artificial rock fracture model	- Water - 4 Silicone oils	NR	Water:1 × 10-3 *1st oil*: 1 *2nd oil*: 0.5 *3rd oil*: 0.1 *4th oil*: 0.05	NR	-	-
Chen 2018 [44]	Artificial rock fracture model	- 3 Glycerol solutions (GS) - Silicone oil (SO)	NR	*1st* GS: 6.5 × 10^−3^ *2 nd* GS: 0.065 *3rd* GS: 0.65 SO: 6.5 × 10^−4^	NR	-	-
Dawson 2012 [45]	Large-scale apparatus and geomechanical experimentation device (LARGE)	- Brine - Corn syrup solution	NR	2 × 10^−3^ 50	NR	0.4	1.97 × 10^−12^

### 4.1 Miscible displacements

As mentioned in Section 3, MVF differentiates from IVF by presenting a more fingered and elongated pattern, even with defined fractal structures, as they are driven not only by viscous but also diffusive gradients. These findings were early reported in smooth Hele-Shaw cells for *2D* displacements [6, 7, 8]. However, more complex experimental setups were made from the standard Hele-Shaw cell to resemble a porous media. One example was to imprint porous patterns on the smooth surface. In this regard, Chen [9, 17, 18] studied radial MVF on standard and modified Hele-Shaw cells. These last ones included porous patterns made by photoetching, which had the purpose of inducing anisotropy to the system emulating porous rocks.

Chen's studies aimed to understand the randomness of the generation of the MVF patterns at different flow rates, porous patterns, viscosity ratios [17], and wall roughness [18]. The results qualitatively described the generation of finger patterns and even proposed correlations that described finger length and pattern density through time. Moreover, a comparison against IVF was made, which will be discussed at the beginning of Section 4.2. The experiments were conducted using water and glycerin [17] and water and dyed water [18]. Several findings were derived from his observations. For example, at different flow rates, the finger pattern stability is almost invariant on smooth Hele-Shaw cells.

On the other hand, on the modified Hele-Shaw cells, at different flow rates, the MVF was chaotic. This meant, for example, that the fingers splitting rate increased at higher flow rates leading to low sweeping efficiencies. The MVF was found to be more severe and increased the fractal nature of the miscible fingers. Finally, on rough surfaces, it was found that the finger length increased with the wall roughness. The results obtained by Chen confirmed the ones previously obtained by Hu [16] in this regard, who previously conducted a very similar study on imprinted cells using water and glycerin.

Chen also developed correlations relating pattern density and finger length as a function of time for smooth Hele-Shaw cells [9]. Measurements of miscible radial displacements were made, especially to study the finger generation through time. A rigorous characterization was made to determine the birth of new MVF generations, which refers to the appearance of new fingers after splitting, and its fractal nature. It was found that the fractal number remained constant after *ten* generations of splitting.

Finding the fractal nature of MVF has also been of interest. This condition was studied by Peters using water and glycerin on unstable displacements [19]. Peters and Chen's [9] studies were about linear and radial displacements, respectively. So, the fractal dimensions achieved were different for both cases despite having worked with the same substances. While Peters reported a fractal dimension between 1.9 and 2 on the linear displacement, Chen reported a fractal dimension between 1.5 and 1.69 on the radial displacement. Additionally, both authors concluded that the injection rate mildly influenced the fractal nature of the displacements.

On other experimental setups, Pope [20] studied the degradation of proppant-pack due to MVF. Proppant-pack is used on hydraulic fractures to hold the fracture while increasing oil mobility. However, these devices suffer from degradation leading to VF and the consequent reduction of the fracture conductivity. The results of this study were related to the variations of mobility ratios, guar gum concentrations, and sweep efficiencies derived from proppant degradation and MVF. As main findings, relationships between reducing porosity to retained permeability and retained permeability to viscosity ratio were obtained. Additionally, the most important finding was the proposal to predict the retained permeability of a fracture with proppant-pack where the damage mechanism is MVF.

The use of sand packs as porous media is also a common experimental approach to study VF. Cuthiell [21] used this setup as a small visual cell in *2D*, to study miscible vertical displacements of heavy oils systems with viscosity ratios up to 100000. Four different experiments were conducted, three with toluene as miscible liquid solvent and one with n-butane as the gaseous solvent. In each experiment, the porous properties of the sand packs and the heavy oil properties were varied. The liquid displacement directions on the sand pack setup were from bottom to top, while the gas ones were from top to bottom. It was found that a single dominant finger characterized the MVF, while the smaller fingers grew slower for both liquid and gaseous displacements. Regarding the efficiency of the liquid-liquid displacements, it was found that despite having used a miscible solvent, the fingering was severe due to the substantial viscosity differences. On the contrary, the gaseous-liquid displacements tend to be more stable due to the rapid coalescence of gaseous fingers achieving better displacements, and hence, a better sweep efficiency than the liquid-liquid displacement.

In more recent research, MVF was studied by Malhotra [14] to analyze the mixing zone in the finger front. The experiments consisted of conducting linear displacements in a standard, or smooth, Hele-Shaw cell for viscosity ratios up to 1225, along with different injection rates for several viscosity ratios. While it has been known that the fingering worsens with the viscosity ratio, it was found that the mixing velocity at the finger front increased significantly for viscosity ratios up to 343. Above this value, the mixing velocity remained constant.

**Figure 7:**
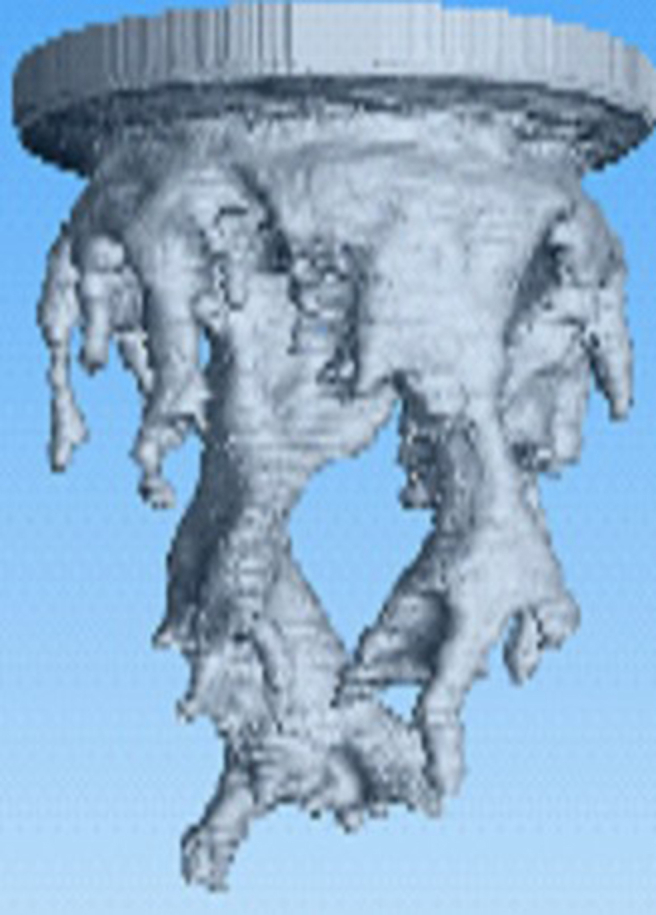
Experimental results of *3D* VF in a cylindrical packed bed. The results were obtained with a micro-focused x-ray tomography scanner. Image adapted from Suekane [22].

Finally, a *3D* study of MVF was made by Suekane using a micro-focused x-ray computed tomography methodology [22]. For this experimental study, a cylindrical packed bed was used as porous media, and *seven* different fluids were considered. The selection of the fluids ensured mobility ratios ranging from 1.3 to 5200, while three different Péclet numbers were also considered to study the effects of the convective to diffusive rates. It was found that the dynamics of MVF growth were very similar to those found in *2D*, sharing features such as splitting, coalescence, and shielding. Additionally, the results allow to determining the solute's concentration profiles, in this case, sodium iodide, in the finger front. It was found that sodium diiodide concentration decreased linearly at the finger front. However, it decreased sharply at the fingertips. Lastly, the experimental results showed that while the dispersion of fingertips was enhanced at high Péclet numbers *≫1*, the mixing increased at high viscosity ratios. An illustration of the obtained results in the *3D* experimental study is shown in Figure 7.

### 4.2 Immiscible displacements

As mentioned in Section 4.1, Chen conducted several studies using the Hele-Shaw cell to understand MVF. However, also conducted several studies on IVF using oil and glycerin [9, 17, 18]. On smooth Hele-Shaw cells, Chen found that the injection flow rate does have a significant incidence on the finger pattern where higher flow rates worsen the fingering as they tend to show higher side branching and tip splitting, contrary to MVF. Moreover, comparing radial and linear Hele-Shaw displacements, it was found that radial displacements had more severe fingering, concluding that these displacements were more unstable as the circular front become more unstable than the linear interface found in linear displacements.

On the other hand, on the modified or imprinted, Hele-Shaw cells, the same chaotic growth was found in MVF, where the finger front was unstable and showed fractal behavior and dendritic patterns well. This meant that in more realistic porous media, it was possible to find fractal nature on immiscible displacements. Nevertheless, at lower flow rates, the fingers tend to compact and become more stable like those found in the smooth cells displacements. In conclusion, the immiscible displacements were more stable than the miscible ones as the interfacial tension stabilized the fingering, even under the induced anisotropy caused by using modified Hele-Shaw cells.

Image processing has been another technique used to study IVF. Peters developed an image processing technique to study IVF in a semi *3D* approach using cross-sections of corefloods [23]. The information obtained by the image processing was mainly about finger lengths, areas, and frequency of the patterns. Also, it helped to understand fingering birth, growth, and population on real porous media. This technique consisted of slicing corefloods to reconstruct the displacements, analyzing the sequence of slices by image processing. This technique was also helpful in the calculation of the Effective Interfacial tension and the Stability Number *(N*_*S*_*)*, which helps to determine if displacement is stable or unstable (*stable if N*_*S*_*<π*^*2*^*)*. Despite this innovative technique, it was slow and inefficient as it required the destruction of the core samples, where the author recommended a nonintrusive or nondestructive approach.

Understanding the birth of the fingers and their splitting dynamics is one of the most critical concerns about this phenomenon. Lajeunesse [24] experimentally studied the dynamics of tips splitting, comparing linear and radial IVF in *2D*. As a result, it was developed a mathematical model that could describe the formation of the fingers. This model began with the calculation of the thresholds of instability when the fingers were born. Derived from these disturbances, the shape of fjords separating the branches (which are parts of a finger) were predicted, and consequently, the stability and shape of the branches. Finally, with the information obtained from the initial disturbances and shape of the branches, the model could determine the fjord length distribution.

IVF is also accounted for in EOR techniques. Some of these usually use non-Newtonian fluids, namely [25, 46] polymer solutions, gels, muds, slurries, among others. Lindner studied two non-Newtonian fluids to determine the influence of their rheological properties on the fingering process [25] in IVF. These two were used as rigid and flexible polymers, both at different concentrations. First, solutions of Xanthan gum were used as rigid polymers. The selection of this polymer was to determine the influence of their strong shear-thinning behavior on the fingering. At low concentrations, it was found that the rheological properties did not have a significant effect on the fingering. Hence, it was assumed that these polymers at low concentrations had a Newtonian behavior during the fingering. Contrarily, at high concentrations, the shear-thinning properties were evident, where fingers became narrower than a Newtonian fluid. Finally, on this kind of fluids, the propagation velocity was different compared to the Newtonian counterpart. Lastly, a model was proposed that could account for these differences. A polyethylene oxide solution was used as the second polymer for this study, cataloged as a flexible polymer. It was found that fingers were more extensive than a Newtonian fluid, and it did not affect the propagation velocity of the fingers. Additionally, it was suggested that the calculation of the Effective Interfacial tension should consider the influence of the large normal stresses usually found in these kinds of fluids.

Another researcher that studied IVF in polymer solutions was Kawaguchi [26]. Two different silica-polymer suspensions and radial and linear displacements in Hale-Shaw cells were considered in this study. For the radial displacements, a shear-thinning suspension made of silica and Hydroxypropyl Methyl Cellulose was used. Also, for linear displacements, a shear-thickening suspension made of silica and Poly Propylene Glycol was used. First, for shear-thinning experiments, the results suggested that the polymer concentration influenced the interfacial instability. Additionally, the author introduced the concept of the modified Darcy's law model, where the viscosity of the suspensions could be defined as a shear-dependent viscosity showing fair agreement with the experimental data of finger velocities. On the contrary, the shear-thickening suspensions showed different behavior. In this case, the fingering instability was associated with the shear rate of the silica suspension. In other words, at the critical shear rate of the silica suspension, it was evidenced the appearance of interface instability and the birth of the first fingers. Lastly, for silica concentrations above 7.5% wt it was found that the modified Darcy's law disagreed with the experimental data as the finger velocities tend to be lower than the predicted ones.

Kawaguchi also studied IVF for emulsified flows in the Hele-Shaw cell [27] to characterize its displacement morphology. In this study, two emulsified fluids were used: i) silicone oil and a solution of Hydroxypropyl Methyl Cellulose (HPMC) with water, and ii) HPMC with water. It was found that the finger pattern was characterized as having crack-like fingers, especially at low injection rates. On the contrary, for large injection rates, the fingers grew with ramified patterns. Finally, a fractal dimension analysis allowed characterizing morphological changes in the fingering growth. For example, it was determined that a fractal number of 1 corresponded to a crack-like pattern, while a value of 1.4 corresponded to cups-like ramified patterns. Lastly, a fractal number of 1.6, the finger morphology corresponded to cups-like branches with thicker ramified trunks patterns.

Li studied air-oil displacements in a radial Hele-Shaw cell to address the dynamics of the finger grown and finger interactions [28]. It was found that for long periods of time, the finger shapes were compact, symmetric, and dependent on a pre-factor of the injection rate. This pre-factor was defined as a dimensionless constant that described the perturbances of the fingers at different injection rates. Finally, a morphology diagram described IVF growth at different injection rates relating to the symmetry of the finger shapes and the injection pre-factor. This diagram is also used for controlling the shape of IVF by controlling the injection rate.

Sinha proposed a different design of the standard Hele-Shaw cell to study the fractal nature of IVF patterns from gas-liquid displacements [29]. While the traditional Hele-Shaw cell consists of two plates at rest, this study's modified setup consisted of lifting the upper plate at constant pressure to allow a concentric displacement. With this technique, it was possible to characterize the different stages of the displacement and their respective finger pattern, associating them with the fractal number through time. On the one hand, it was found that the fractal number increased linearly with the lifting pressure applied to the upper plate. On the other, at constant lifting force, the fractal number through time has a concave downward profile, where a sharp increase characterized the first seconds of the displacement, indicating the birth and initial propagation of the fingers; In contrast, a consecutive rapid decrease of the fractal number indicated the fingering propagation. Finally, it was found the presence of cavitation at high lifting pressures (>22500 *kg/m*^*2*^).

Another experimental approach to study IVF in *2D* is by using micromodels. This approach is more realistic to describe the flow through porous media than the Hele-Shaw cell, mainly because properties such as porosity and permeability now exist and can be defined according to the porous pattern's arrangement micromodel. Jamaloei used this experimental approach to study multiphase flow through porous media. Moreover, its studies about IVF focused on the displacement of Heavy Oil [31, 32], polymer flooding [30], and surfactant injection [32] using existing injection schemes (five-spot injection) rather than linear displacements, attempting to replicate actual production conditions of an oil field at laboratory scale.

**Figure 8:**
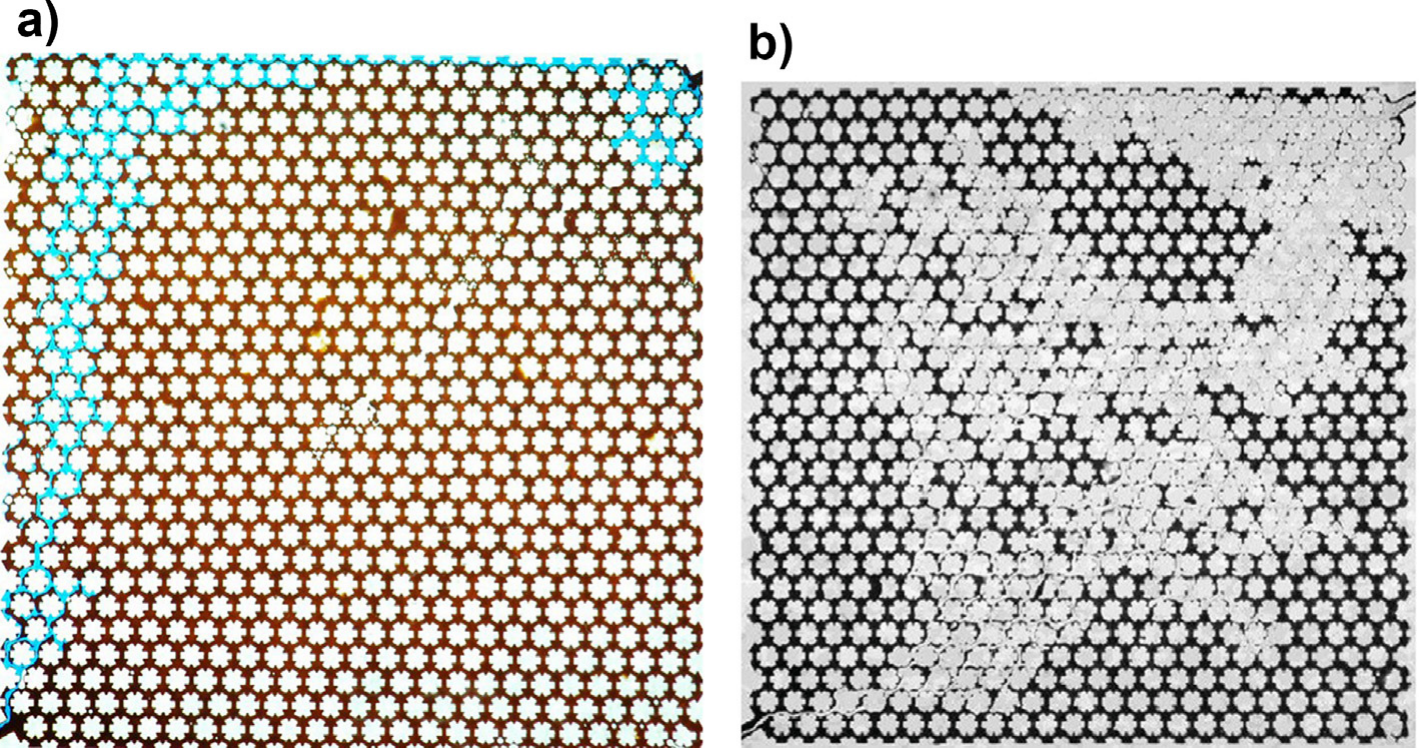
Example of a micromodel with a defined porous pattern. a) VF study under Waterflooding and b) VF under Polymerflooding. Images adapted from Jamolei [31, 47].

Regarding the study of Waterflooding, Jamaloei [31] focused on the experimental analysis of fingers length, width, growth rate, population, and frequency of instabilities. In this study, water injection attempted to emulate a five-point injection scheme on the micromodel, replicating a common production scheme at a lab scale. It was found three different regions or stages of IVF: i) Microfingers at early stages, ii) Fingers and channeling at the edges of the domain, and iii) Peripheral frontal advance until breakthrough. It was also found that in systems with high interfacial tensions >8.4 *mN/m*), the number of fingers is independent of the flow rate. On the contrary, for low interfacial tensions (<0.0065 mN/m) occurred the opposite. An exemplification of the micromodel experimental setup is illustrated in Figure 8.

On the other hand, for the polymer flooding study, Jamaloei used oil and a Xanthan gum solution [30]. In this case, the results were different from the previous study. First, the displacement was different; even three utterly different IVF regions or stages were identified: i) Microfingers at early stages with diagonal displacements, not sideways, ii) Spreading, with sideway growth, and iii) front channeling and breakthrough. Also, it was found that for Low Tension polymer floodings, the number of fingers depended on the injection rate, where an increase in the injection rate caused a decrease in the population of fingers.

Finally, Jamaloei studied IVF associated with alcohol-assisted surfactant waterflooding [32]. A new micromodel setup was used in this case. While the previous studies used a micromodel with a homogeneous and predetermined porous pattern, this last one replicated a sandstone sample. Therefore, the new porous pattern was heterogeneous, being more realistic. Under this recovery technique, three distinctive regimes were identified: i) Early displacement, ii) Breakthrough and early post-Breakthrough, and iii) post-Breakthrough to late displacement. This classification was defined based on the behavior of the pressure field, pressure drop across the porous medium, finger growth, and population. Also, this flooding technique tend to generate micro fingers caused by the low interfacial tension (0.0065 *mN/m*) and by capillary forces. The most noticeable characteristic of this kind of fingering was that the fingers tend to spread independently, avoiding the contact and coalescence between them.

Heterogeneous micromodels were also used by Pei [33, 34], who studied IVF in alkaline flooding for Heavy Oil recovery. In his first study, the alkaline fluids used were sodium carbonate and sodium hydroxide [33]. Additionally, the micromodel used in this study was a replica of a porous section of a core. It was found that alkaline flooding at a microscopic scale reduced IVF as it lowered the interfacial tension, which tends to form water drops in the oil phase mitigating IVF and the channeling phenomenon. Additionally, higher concentrations of the alkaline solution allowed faster penetration. Consequently, a better sweep efficiency was achieved under these conditions than waterflooding experiments that channeled the flow into the main finger.

**Figure 9:**
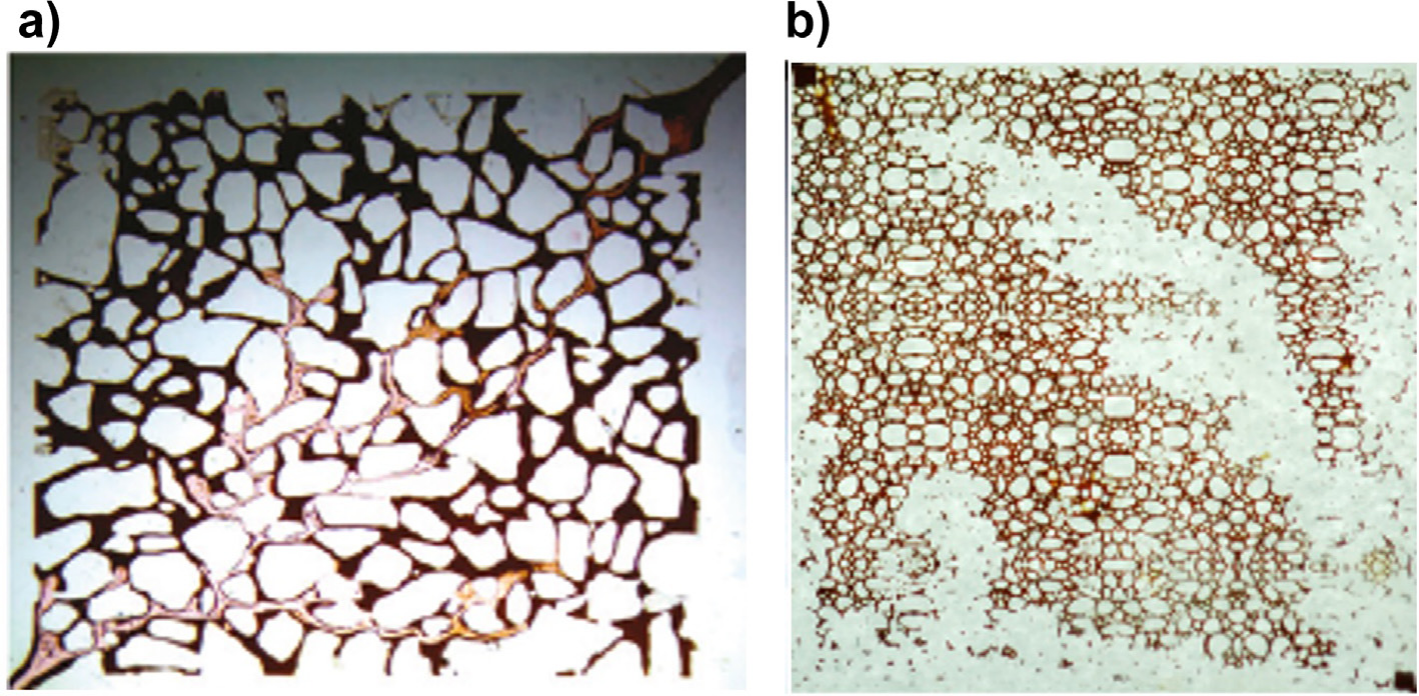
Heterogeneous porous pattern micromodels. a) Replica of a cross-section of a core, adapted from Pei [34]. b) replica of a sand pack porous pattern, adapted from Jamaloei [39].

Pei's second study considered two experimental setups: i) A sand pack and ii) a micro model [34]. Additionally, two displacement fluids were used: i) Alkaline and ii) alkaline-surfactant chemicals, using SLPS as a surfactant. First, the micromodel was used to characterize the IVF using both kinds of fluids. On the one hand, it was found that the alkaline displacements reduced IVF as this fluid tends to generate water-in-oil droplets improving sweep efficiency compared to its alkaline-surfactant counterpart, at least from a qualitative point of view from image processing. On the contrary, the alkaline-surfactant fluid caused the opposite reaction where oil-in-water droplets were formed, failing on its purpose to reduce IVF at getting ultra-low interfacial tensions. Finally, the preliminary observations found in the micromodels were confirmed with sand pack experiments where the alkali flooding achieved a better oil recovery than its alkaline-surfactant counterpart by 3–8%. Nevertheless, the author argued that alkali-surfactant flooding could improve using an appropriate surfactant, i.e., one that allowed the formation of dispersed droplets. Some illustrations about heterogeneous micromodels are presented in Figure 9.

Further studies of alkaline floodings were made by Doorwar, who studied the dynamics of alkali-surfactant flooding [35]. The experiments were conducted using a micromodel setup and three different oils: one light oil and two heavy oils. Additionally, water, HPAM, and alkali-surfactant were used as flooding fluids. Two different sets of experiments were conducted: Secondary (water and polymer injection) and tertiary (alkali-surfactant injection) flooding, along with the numerical modelling of the IVF using the Diffusion Limited Aggregation (DLA) model. Regarding the secondary recoveries, it was found that displacements at increasing viscosity ratios increased the severity of the fingering, even showing a fractal-type front. Later, during the tertiary floodings, the micromodels saw that the injection of chemicals improved oil recovery by better sweeping along with the domain. Finally, it was determined that simulations based on the DLA model could resemble the displacements and IVF obtained from the experiments.

Doorwar developed further studies using micromodels to propose and validate a *1D* model capable of describing immiscible linear displacements under unfavorable viscosity ratios accounting for the IVF phenomenon [36, 37]. The model was validated against experimental data obtained from corefloods, showing fair agreement. Finally, the micromodel experimental setups used were made of glass and silica, while several injection rates and ten different mobility ratios, up to 10500, were considered. The experiment results obtained from the micromodels showed that the fingering increased at increasing mobility ratios, even achieving fractal behaviors. On the other hand, it was found that low injection rates favored the growth of wide fingers, increasing sweep efficiency. These results were also confirmed with coreflood experiments. Also, this study proposed a new dimensionless number that correlated the capillary and viscous forces, which allow determining that viscous effects are more substantial than the capillary ones for oil recovery. Finally, a lumped finger model validated against experimental data was developed to determine pseudo relative permeability curves. The author argued that this model could simulate immiscible displacements capturing IVF without the need for fine grids.

Zhang made other studies based on micromodels for IVF [38, 39]. In Zhang's first study, two different wetting and four non-wetting fluids were used [38]. An analysis of the influence of viscous and capillary forces over immiscible displacements was made at different injection flow rates. The wetting fluids, water, and polyethylene glycol were paired with the non-wetting fluids during the experiments to obtain viscosity ratios of up to 4 orders of magnitude. Finally, the displacements were studied using fluorescent microscopy. Two kinds of fingering mechanisms were found depending on the wetting phase: i) VF found on polyethylene glycol displacements, and ii) Capillary Fingering (CF) on water displacements. Finally, while CF was found to happen at low Capillary Numbers (Ca), mainly (log *Ca < −3*), it was also found that water stable displacements could occur at high viscosity ratios and Capillary numbers (*log Ca > −3 and log M > 0*).

Zhang's second study was about the displacement of water by liquefied *CO*_*2*_, concerning to *CO*_*2*_ sequestration [39]. This study considered a heterogeneous porous media made of two different permeability zones. First, it was found that *CO*_*2*_ displacements were always unstable for a wide range of injection flow rates. Moreover, it was found that CF tends to happen in the high permeability zone at low flow rates, while VF was dominant at high injection rates. On the contrary, at the low permeability zone, only VF was evidenced. Also, in this study, it was proposed a model capable of predicting the *CO*_*2*_ saturation as a function of the injection rate. Despite the model was reliable at low flow rates, it tended to fail at high injection rates. It was argued that this lack of reliability occurred because the model could not account for the transition from CF to VF. Finally, it was found that the interfacial area during *CO*_*2*_-water displacements tended to increase at higher *CO*_*2*_ saturations, for both high and low permeable zones.

Another study about *CO*_*2*_ VF on micromodels was made by Wang & Zhang, expanding the previous work [40]. In this research, it was studied the injection of supercritical *CO*_*2*_ at water reservoir conditions. Two injection methods were evaluated: discontinuous and continuous injection rates. It was found that the CF was dominant at most injection rates for the discontinuous case, while the VF only occurred at the highest injection rates. However, at continuous injection rates, both mechanisms tend to happen. Finally, it was found that it was possible to achieve a high saturation of *CO*_*2*_ at low injection rates, especially for the discontinuous case.

Sharma also conducted a micromodel study, using a sandstone pattern, for oil-water displacements using three different oils [41]. The purpose was to demonstrate that for unstable immiscible displacements, the Darcy-based modelling was not advisable. It was found that the saturation profiles along time did not have similar behavior to the Buckley-Leverett theory, except for low viscosity oils. Therefore, it was determined that unstable displacements do not scale linearly with position/time, which meant that capillary-dominated displacements should not be modeled using equilibrium models, like those found in reservoir simulators. Also, it was suggested that non-equilibrium models should account for saturation history.

Zhao made the last experimental micromodel study reviewed to determine the effect of wettability on unstable displacements [42]. Moreover, the micromodel findings were contrasted and complemented with coreflood experiments. In this study, six different cores and one micromodel setup were used, the last one to visualize the effects of wettability during the displacement. Also, it was not only studied the effect of wettability on the fingering and permeability and flow rate effects. Derived from the experimental results, two correlations were proposed to model strongly oil-wet and water-wet recoveries. Also, it was found that IVF was severe on oil-wet systems, specifically at low permeabilities for water-wet systems.

IVF in rough fractures has also been addressed. Chen conducted different studies in this regard [43, 44]. The first research was focused on the study of the crossover from CF to VF [43]. The experiments consisted of water displacing oil for seven flow rates and four viscosity ratios. It was found that at the crossover, the competition between viscous and capillary forces causes a saturation reduction of the displacing fluid. A change in the fingering dynamics explained that this reduction at the crossover were narrower and fewer fingers appeared. Finally, it was proposed a flow pattern diagram that described if the displacement was dominated by CF, VF, crossover, or on the contrary, a stable displacement.

**Figure 10:**
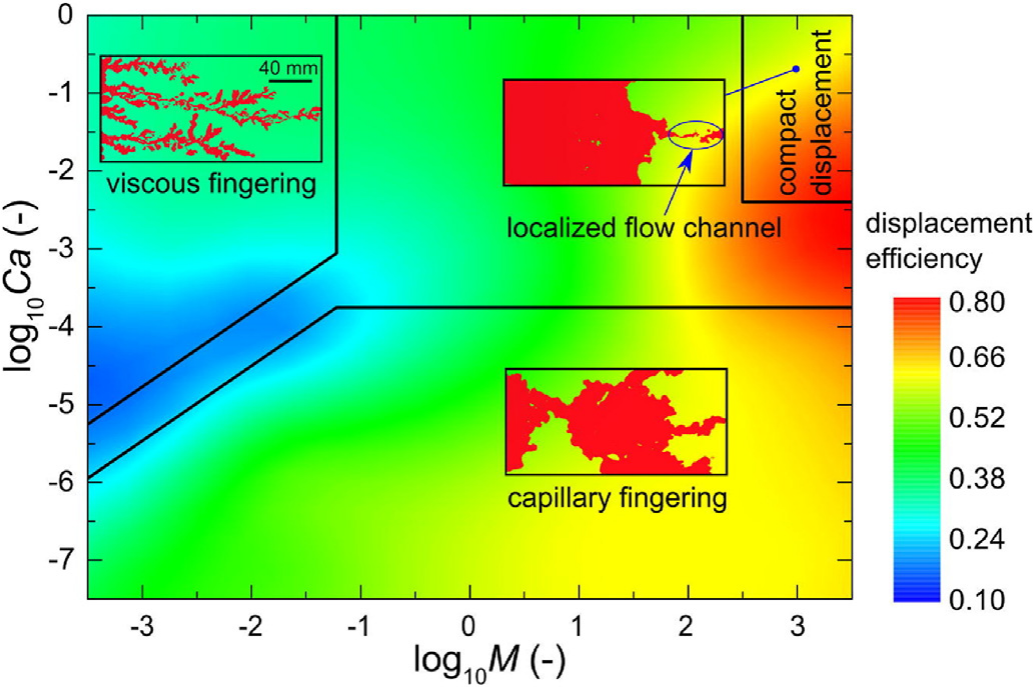
Flow pattern map on fluid displacement morphologies developed by Chen. Adapted from Chen [44].

Chen's second experimental study focused on constructing the phase diagram on rough fractures, especially to identify [44]. As mentioned before, the phase diagram described the displacement process: VF, Capillary Fingering, stable displacement, and crossover. On the one hand, on the characterization of the crossover from CF to compact displacement, it was found that this event occurred at Ca numbers between −3.25 < log_1*0*_ Ca < −2.55, where the highest sweep efficiency occurred at log_10_ *Ca = -2.25*. On the other hand, from the analysis of the crossover from VF to compact displacement, it was determined that the crossover occurs between 2 < log_10_ *M* < 3, being at log_10_ *M* = 3 when compact displacement occurs. However, it was found that the displacement efficiency decreased with an increase of the Ca number when the regime is in the compact displacement region. The author explained that this behavior was due to the dynamics of the flow at the fracture aperture where the influence of the geometry during the displacement process had a strong effect, especially where the surface roughness tends to affect the contact angle of the immiscible displacement. This study, along with the previous study results about unfavorable displacement conditions, helped develop a flow pattern map that predicted the occurrence of localized flow at different injection rates on rough fractures. An illustration of this map is shown in Figure 10.

Dawson conducted an innovative study of VF with a *3D* setup for displacements with mobility ratios up to 25000 [45]. The experimental device was a chamber of 3.5*m* in diameter and 4*m* high, capable of testing porous materials of 2.1*m* in diameter and 0.5*m* high. This chamber was also capable to achieve pressures up to 2100*psi* and temperatures ranging from -30 to 90 °C. Also, it could work on injection or production rates up to 200 *cm*^*3*^*/min*. This experimental setup allowed to establish two different methods to study *3D* displacements and VF: i) measurements via data acquisition through time, and ii) visual inspection using an excavation device. This device allowed to reproduce VF in *3D* and helped on the data acquisition to develop numerical models capable of simulating VF. Finally, VF has also been studied on setups with variable boundaries. In this regard, McCue studied a modified Hele-Shaw cell that had an elastic membrane instead of the classic top plate [48]. In this study, oil was displaced by air. From this modified Hele-Shaw cell it was found new kinds of fingers or finger patterns. These new patterns were described as: sideways oscillating, “dentric,” and “molar” like teeth.

## 5 Modeling of VF at the microscale: *2D* and *3D* simulations

The previous section focused on experimental studies about VF. Most of them using Hele-Shaw cells and other sophisticated experimental setups that have allowed the understanding of this phenomenon. However, the development of numerical models capable of simulating VF has also been significant. In this regard, many efforts have been made not only to predict these phenomena but also to account for or consider their effects on operational variables such as sweep efficiency, pressure drop, front velocity, and recovery rates. This section reviews the studies made on the modeling of VF, mainly at the microscale. Additionally, the summary of the codes used and fluid properties used in these studies are summarized in Tables 3 and 4.

**Table 3:** Summary of the numerical setups and fluids used for the modeling of miscible displacements.

Author	Model/Code used	Fluids	Density $\left(\frac{kg}{m^{3}}\right)$	Viscosity (*P α s*)	Porosity	Permeability (*m*^2^)
Odeh 1988 [49]	Finite element/In-House	- Solvent - Oil	RTM	RTM	0.2	2.66 × 10^−12^
Fayers 1988 [50]	Finite element/In-House	- Solvent - Oil	RTM	RTM	-	-
Araktingi 1990 [51]	Particle tracking/In-House	- Solvent - Oil	NR	RTM	NR	9.86 × 10^−12^ 4.93 × 10^−13^ 9.86 × 10^−14^
Araktingi 1995 [52]	Particle tracking/In-House	- Solvent - Oil	NR	RTM	NR	RTM
Ku 1989 [53]	Adaptive Pseudospectral Matrix Element/In-House	- Solvent - Oil	NR	RTM	NR	RTM
Brock 1991 [54]	Finite element/In-House	- 2 Oils - 2 Solvents	*1st oil*: 836 *2nd oil*: 854 *1st solvent*: 854 *2nd solvent*: 836	*1st oil*: 0.023 *2nd oil*: 0.046 *1st solvent*: 5.8 × 10^−4^ *2nd solvent*: 5.8 × 10^−4^	0.836 0.854	RTM
Moissis 1993 [55]	Galerkin/In-House	- Solvent - Oil	NR	RTM	0.2	RTM
Blunt 1994 [56]	Finite element/In-House	- Gas solvent - Oil	NR	1.16 × 10^−5^ 1.03 × 10^−3^	NR	RTM
Ferer 1993 [57]	Square Lattice model/In-House	- Hypothetical displacing fluid - Hypothetical displaced fluid	NR	RTM	NR	RTM
Tang 1996 [58]	Diffusion Limited Aggregation Model/In-House	- Water - Glue	NR	NR	-	-
Yang 1998 [59]	Finite element/In-House	- Hypothetical displacing fluid - Hypothetical displaced fluid	NR	RTM	-	-
Manickman 1993 [60], 1994 [61], 1995 [62]	Hartley spectral/In-House	- Hypothetical displacing fluid - Hypothetical displaced fluid	NR	RTM	-	-
Coutinho 1999 [63]	Finite element/In-House	- Hypothetical displacing fluid - Hypothetical displaced fluid	NR	RTM	-	-
Mishra 2010 [64]	Finite element/In-House	- Hypothetical two-component displacing fluid - Hypothetical two-component displaced fluid	NR	RTM	-	-
Islam 1999 [65]	Finite element/In-House	- Water - Glycerin	1000 1085	0.0001003 1.49	0.39	2.52 × 10^−8^
Saghir 2000 [66]	Finite element/In-House	- Water - Glycerin	1000 1085	0.0001003 1.49	0.39	2.52 × 10^−8^
Naami 1999 [67]	Finite element/In-House	- Water - Glycerin solutions	NR	RTM	0.39	2.52 × 10^−8^
Islam 2006 [68]	Hartley spectral/In-House	- Hypothetical displacing fluid - Hypothetical displaced fluid	NR	NR	-	-
Sajjadi 2012 [69]	Spectral/In-House	- Hypothetical displacing fluid - Hypothetical displaced fluid	NR	NR	NR	RTM
Mcdowell 2016 [70]	TOUGH2/Comercial	- Water (at several temperatures) - Oil	NR	RTM 9.98 × 10^−4^	0.3	0.1 × 10^−12^
Guan 2003 [71]	Finite element/In-House	- Hypothetical displacing fluid - Hypothetical displaced fluid	NR	RTM	-	-
Ghesmat 2008 [72]	Pseudo-spectral/In-House	- Hypothetical displacing fluid - Hypothetical displaced fluid	NR	RTM	-	-
Sesini 2010 [73]	Galerkin/In-House	- Hypothetical displacing fluid - Hypothetical displaced fluid	NR	RTM	-	-
Li 2015 [74]	Galerkin/In-House	- Hypothetical displacing fluid - Hypothetical displaced fluid	NR	RTM	0.2	3.72 × 10^−13^
Becker 2018 [75]	Galerkin/In-House	- Hypothetical displacing fluid - Hypothetical displaced fluid	NR	RTM	NR	2 × 10^−12^
Moortgat 2016 [76]	Galerkin/In-House	- Oil - *CO*_*2*_ - Water	736/818 731/647 NR	1.28/1.09 (× 10^−3^) 6/9 (× 10^−5^) 4.8 × 10^−4^	0.13	RTM
Hamid 2018 [77]	Finite element/In-House	- Solvent - Oil	NR	RTM	-	-
Nijjer 2018 [78]	Finite element/In-House	- Brine - *CO*_*2*_	NR	RTM	0.3	RTM

**Table 4:** Summary of the numerical setups and fluids used for the modeling of immiscible displacements.

Author	Code used	Fluids	Density $\left(\frac{kg}{m^{3}}\right)$	Viscosity (*P α s*)	Interfacial tension (*N*/*m*)	Porosity	Permeabilit $\text{y}\left(m^{2}\right)$
Claridge 1983 [79]	Todd, Dietrich & Chase Inc (RTM)/Commercial	- Oil - *CO*_*2*_ - Water	1000 RTM 1000	RTM	NR	0.29	1.58 × 10^−9^
Zhang 1998 [80]	Diffusion Limited Aggregation Model/In-House	- Oil - Water	NR	NR	NR	NR	NR
Medici 2007 [81]	Pore Network/In-House	- Hypothetical displacing fluid - Hypothetical displaced fluid	NR	RTM	1	-	-
Regaieg 2017 [82]	Pore Network/In-House	- Oil - Water	NR	RTM	NR	0.24	2.46 × 10^−11^
Tian 2015 [83]	Diffusion Limited Aggregation Model/In-House	- Oil - *CO*_*2*_	NR	1.4–1.5 × 10^−3^ RTM	NR	0.2	RTM
Nakayama 1998 [84]	Finite element/In-House	- Air - Water	1.25 998	1.84 × 10^−5^ 1 × 10^−3^	7.275 × 10^−2^	-	-
Belotserkovskaya 2010 [85]	Finite volume/In-House	- Hypothetical displacing fluid - Hypothetical displaced fluid	NR	RTM	NR	NR	NR
Dong 2010 [86]	Lattice Boltzmann/In-House	- Hypothetical displacing fluid - Hypothetical displaced fluid	1 1	RTM	RTM	-	-
Dong 2011 [87]	Lattice Boltzmann/In-House	- Oil - Glycerin solution	877 1210	RTM	RTM	-	-
Shi 2014 [88]	Lattice Boltzmann/In-House	- Hypothetical Newtonian fluid - Hypothetical non-Newtonian fluid	RTM	RTM	RTM	-	-
Wang 2019 [89]	Lattice Boltzmann/In-House	- Hypothetical Newtonian fluid - Hypothetical non-Newtonian fluid	RTM	RTM	RTM	-	-
Shiri 2018 [90]	Lattice Boltzmann/In-House	- Hypothetical displacing fluid - Hypothetical displaced fluid	RTM	RTM	RTM	-	-
Wei 2018 [91]	Lattice Boltzmann/In-House	- Oil - Water - Surfactant	RTM	RTM	RTM	-	-
Yamabe 2014 [92]	Lattice Boltzmann/In-House	- Water - *CO*_*2*_	994 994/668 (RTM)	5.51 × 10–3/5.51 × 10^−4^ (RTM) 5.21 × 10^−4^/5.21 × 10^−5^ (RTM)	0.04	-	-
Soltanian 2017 [93]	Galerkin/In-House	- Water - *CO*_*2*_	981/987 (RTM) RTM	RTM	NR	RTM	RTM
Mostaghimi 2015 [94], 2016 [95]	Finite volume/In-House Eclipse 100/Commercial	- Oil - Water	NR	0.1 0.001	NR	0.205	3.72 × 10^−13^
Adam 2017 [96]	IC-FERST/Commercial	- Oil - Water	NR	RTM	NR	0.205	3.7 × 10^−13^
Lagreé 2016 [97]	GERRIS/Open Source	- Oil - Water	RTM	RTM	NR	-	-

### 5.1 Modeling of miscible flows

Odeh developed an early model to describe miscible displacements in *1D* accounting MVF [49]. The model was based on variables such as the effective viscosity, density, and saturation of the displacing fluid. This model was validated against experimental data for linear miscible displacements showing fair agreement. Also, the model could predict the changes of viscosity and density of the displacing fluid below the critical saturation, which used to be an issue in the modeling of miscible displacements at that time.

An early study on the modeling of *2D* MVF was made by Fayers, who proposed a model which accounted for the effects of gravity [50]. The model, which was validated against experimental data, had the purpose of helping validate an empirical correlation developed by the same author. The model was based on the finite-element method using an in-house code. It was found that the model could describe the MVF showing fair agreement with experimental data. Moreover, the model and the empirical correlation could predict concentration profiles, finger width, flow rates, pressure drops, among other variables in coreflood displacements.

Araktingi conducted a numerical study for displacements accounting for gravity on vertical flows and different porous media, considering homogeneous and heterogeneous porous media [51]. This last one was made of three layers and used two permeability distributions: descending and ascending horizontal layers with 1000, 500, and 100 *md*. First, the model could describe gravity-driven tongues and MVF, having a fair agreement with experimental results. Regarding the displacement efficiency, the homogeneous case showed higher sweep efficiency for high viscosity to gravity ratios than the heterogeneous cases. Nevertheless, when the most permeable layer was at the top for the heterogeneous cases, better sweep efficiency was achieved. However, for “moderate” viscosity to gravity ratios, better sweep efficiencies were achieved when the most permeable layer was at the bottom. Finally, the model was validated against experimental data, at least for the homogeneous case.

Also, Araktingi focused on the modeling of miscible displacements in heterogeneous porous media [52]. The purpose was to determine the effects of the heterogeneity field on MVF growth dynamics. Additionally, the model was compared against experimental data and two *1D* analytical models for validation and comparison at field scale. The experimental results showed that large heterogeneity contrasts had a significant effect on the dynamics of MVF, where they tend to go through the path of less resistance. However, on slight heterogeneity variations, the dynamics were close to those found in homogeneous media. On the other hand, the numerical model resembled the experimental results for homogeneous and heterogeneous displacements for different Pe numbers with negligible numerical dispersion.

Different numerical techniques have been used to improve the modeling of MVF, reducing computational costs and improving the quality of the results. Ku made a study using the Pseudospectral Matrix Element Method to model miscible displacements at high mobility ratios, up to 100, in a five-spot injector-producer pattern [53]. This method allowed to solve the incompressible governing equations in a coupled manner, reducing the number of iterations needed to solve the pressure field and reduce computational cost. In this regard, the author used the Schwarz Alternating Procedure to solve the pressure field. Lastly, the qualitative results showed that the model could describe miscible displacements in five-spot injector-producer well schemes. They kept a reliable description of MVF and an accurate calculation of concentration profiles at different mobility ratios.

Another study in heterogeneous and homogeneous porous media was made by Brock comparing numerical simulations and experiments in packed glass beads in *2D* [54]. In this work, three different flow rates and mobility ratios were considered. Additionally, the fluids used were: two different mineral oils, refined isoparaffin, and toluene. The experimental results showed that the finger growth dynamics in the homogeneous porous media were sensitive to the mobility ratio but not to the studied flow rates. On the contrary, in the heterogeneous field, the fluids flow through the high permeability region. On the other hand, the numerical model could reproduce the experimental results with high fidelity. MVF dynamics at different mobility ratios and flow rates were successfully emulated.

Moilssis also studied the effects of the heterogeneous permeability field on MVF, especially on gravity tongue [54], a main dominant finger that appears due to the density gradients and gravity effects. The code used was based on the Finite Element Method (FEM). It considered oil and a solvent at two viscosity ratios, 10 and 41. It was found that the displacement was dominated by both MVF and gravity tongue, this last one mainly at large density differences. For gravity tongue-dominated systems, it was determined that the recovery efficiency was drastically reduced after the breakthrough. Additionally, the model could predict that tiny fingers tend to interact, merging into main active fingers or channels at the beginning of the displacement.

The proposal of compositional displacement models and high-resolution numerical simulations of compositional displacements were studied by Blunt [56]. A theoretical model capable of accounting for MVF in unstable compositional displacements was developed based on a fractional flow formulation. Although the model neglected capillary and gravitational effects, it could simulate compositional displacements and accurately calculate variables such as concentration profiles. Moreover, the model was validated and compared against high-resolution compositional simulations showing fair agreement. This high-resolution model was also capable of simulating IVF; however, it restricted low-pressure systems with a mild prediction of IVF. On the contrary, it was capable of predicting severe fingering from high-pressure vaporizing and condensing injections.

Regarding the modeling of the MVF fractal nature, Ferer conducted one of the very first numerical studies on this regard in *2D* [57]. The purpose was to compare the conventional reservoir simulators, which assumed that the saturation front was lineal, against simulations where the saturation front was fractal. The simulations used in this work were based on the Square Lattice method using an in-house code. Also, the model predicted fractional flow as a function of saturation and time, contrary to the reservoir simulators. It was found that the fractal behavior occurred at viscosity ratios larger than 10000. Furthermore, the author argued that fractal MVF could happen early for the evaluated mobility ratios at the reservoir scale. After that, it breaks away, showing a linear or stable displacement.

Yang addressed VF in *2D* displacements in systems with no-flow boundaries [59]. The purpose of this work was to simulate and understand why the fingers tend to propagate faster in no-flow boundaries or walls compared to fingers at the interior of the domain under transverse equilibrium. It was found that MVF was severe on the walls at increasing mobility and aspect ratios but diminished at increased permeability disorders. This behavior was explained by the existence of slip-conditions at no-flow boundaries on models based on Darcy's law rather than numerical instability. However, the author suggested further study of this phenomenon, arguing the usage of Brinkman's law instead of Darcy's law.

Another field of study on miscible displacements has been with systems with non-Monotonic Viscosities. Here, the viscosity variation at different ranges of concentrations does not necessarily mean that the peak viscosity is achieved at the highest concentration phases. On the contrary, it can be achieved at intermedium concentrations [98]. Manickam developed several studies to address the modeling of miscible displacements with nonmonotonic viscosity profiles [60, 61, 62].

Manickam's first study used a numerical model to predict the fluid flow in porous media with nonmonotonic viscosity profiles [60, 61]. The model was capable of simulating this kind of displacement and described the effects of physical dispersion. This allowed simulating the viscosity profiles at several ranges of concentrations. Additionally, the results showed that even at early stages, the diffusion effects did not allow the flow to stabilize, contradicting the concept that diffusion usually helps stabilize the flow. Consequently, a physical mechanism that explained the diffusion effects on the stability of the displacement through time was proposed.

In Manickam's second work, MVF under nonmonotonic displacements was studied. A numerical model was developed to compare MVF on monotonic and nonmonotonic viscosity profiles. It was found that the dynamics of MVF were different. First, the nonmonotonic displacements had the singularity of presenting reverse fingering. This caused the displaced fluid to penetrate the displacing fluid; in other words, it traveled in the opposite flow direction. Because of this phenomenon, forward and reverse mixing lengths were defined to characterize the fingering in both directions, which also tend to grow linearly. Due to these results, a physical mechanism was proposed to describe the dynamics of forward and reverse MVF in these kinds of displacements.

**Figure 11:**
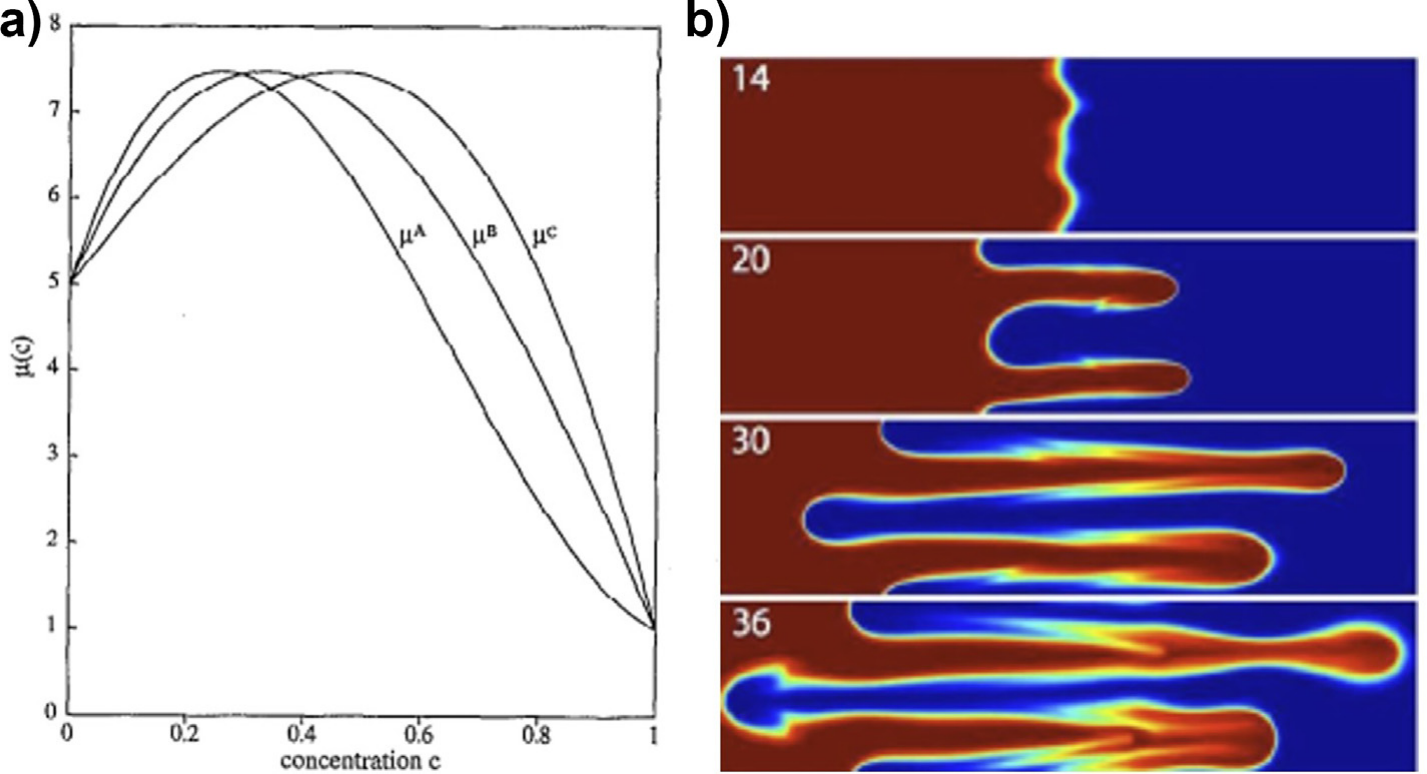
VF at nonmonotonic viscosity displacements. a) Non-monotonic viscosity profile, adapted from Manickam [60]. b) VF at different times illustrating reverse fingering, adapted from Mishra [64].

Finally, the third study of Manickam was about MVF in vertical displacements under nonmonotonic viscosity profiles [62]. The study was made by linear stability analysis and numerical simulations. It was found that the critical velocity changed at different concentrations. Moreover, two displacement regions appeared on nonmonotonic displacements: i) a stable-unstable region and ii) an unstable-stable region. Each region was found to be dependent on the fluid properties profiles and flow velocities. Also, it was evidenced by the presence of reverse fingering in the vertical direction. Finally, it was found that finger propagation had no preferences under linear density profiles. However, at induced viscosity profiles, stable regions were found to avoid reverse fingering. Some results that illustrate the nature of nonmonotonic displacements are shown in Figure 11.

Another researcher who studied VF under nonmonotonic viscosity profiles was Coutinho using FEM [63]. The purpose of this research was to propose numerical techniques capable of boosting MVF numerical simulations. Several approaches were considered to develop adaptive time-steps and iterations per time-step that reduced the computational costs. The developed model could simulate miscible displacements, including anisotropic dispersion, nonmonotonic viscosity profiles, and heterogeneous porous media. It was found that the numerical simulation successfully described MVF at reduced computational costs.

So far, miscible displacements were studied using just two miscible fluids. Mishra addressed displacements of fluids made of two components, each one studying the diffusive effects of having more than two miscible components [64]. This was referred to in Mishra's work as double-diffusion, which also causes nonmonotonic viscosity profiles. Moreover, the two components had slow and fast diffusing properties, respectively. The numerical model used to conduct this study was based on a pseudospectral FEM using a Hele-Shaw cell and different injection rates. It was found that three different MVF regimes characterized these displacements: i) Double diffusive, ii) slow-diffusing component destabilizing, and iii) fast-diffusing component destabilizing. This characterization was made from the fact that the MVF dynamics were different between them. Additionally, the study highlighted the presence of reverse fingering and the appearance of mushroom-like finger patterns.

MVF was simulated based on the momentum, energy, and conservation equations by Islam and Saghir [66, 68] for thermal displacements in *2D*. Here, the momentum equation was based on Darcy's law including the Brinkman term. The developed model not only accounted for a double-diffusive condition, given by the heat and mass transfer terms, but also considered the effects of gravity. Glycerin was displaced by water in a porous media, and the model was validated against experimental data, at least for the isothermal condition. It was found that for isotropic and isothermal conditions, the fingering propagated faster in the middle section. For the thermal displacement, it was found that finger propagation and shapes were not affected. However, the displacement front traveled slower due to the buoyancy effects that acted against the fingers. This buoyancy appeared by decreasing the density of the displacing phase, caused by the higher temperatures used in the thermal displacement. In a separate study, the numerical model developed by Islam and Saghir was improved by Naami [67]. In Naami's work, the numerical scheme used to solve the system was improved by directly solving the partial differential equations.

Another study that addressed thermal displacements was conducted by Islam [68]. In this research, non-isothermal miscible displacements were simulated in a Hele-Shaw setup considering a wide range of solutal and thermal Pe numbers, along with different Lewis (Le) numbers while including the viscosity dependence on concentration and temperature. The model developed was capable of simulating this highly non-linear problem, describing MVF dynamics in thermal displacements. Additionally, it was found that the displacement front was unstable at large Lewis numbers, with the same tendency on the thermal front. Finally, it was possible to establish that the fluid front was more unstable than the thermal front.

**Figure 12:**
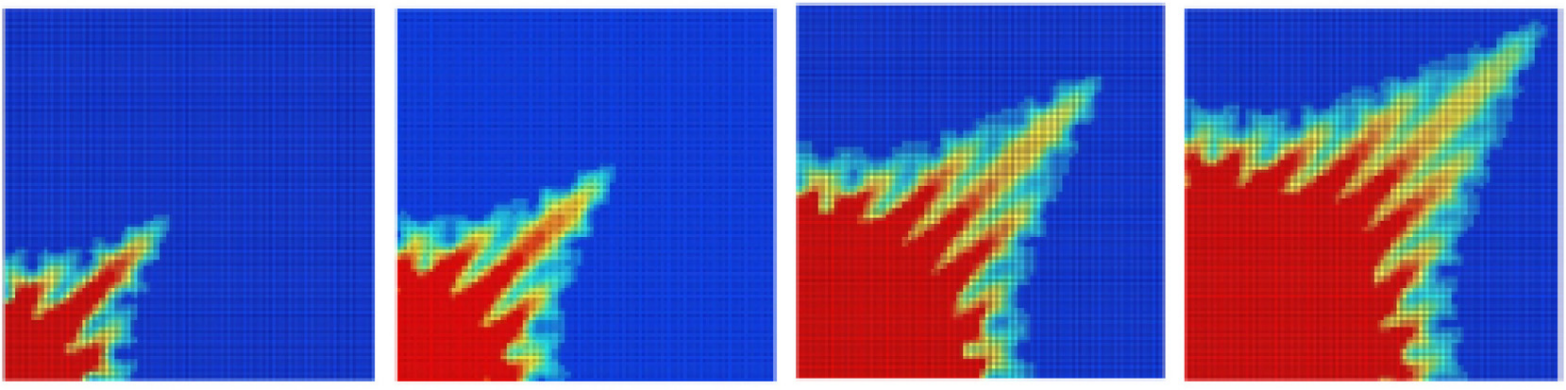
VF on thermal displacements. Thermal front at different times, adapted from Mcdowell [70].

The effects of heterogeneous porous media in thermal displacements were addressed by Sajjaid [69]. In Sajjaid's work, different mobility ratios were considered non-isothermal conditions and simulations on homogeneous porous media. The results suggested that isothermal and non-isothermal displacements in heterogeneous porous media favored channeling. Reduced sweep efficiency and earlier breakthrough times were found. On the other hand, in non-isothermal displacements at homogenous and heterogeneous porous media, the breakthrough times and sweep efficiencies were also reduced at increasing thermal lags. Finally, the model could predict the changes in fingering dynamics between isothermal and non-isothermal displacements, even accounting for thermal lag effects. Some results that illustrate MVF on thermal displacements are presented in Figure 12.

Mcdowell determined that the nature of thermal displacements will always be an unstable event at low injection rates and several injection temperatures for geothermal injections [70]. This conclusion was derived from a linear stability analysis of thermal displacements and supported with numerical simulations. From the linear stability analysis, it was found that at injection temperatures up to 10^2^ MVF is likely to happen even at Pe numbers below the Critical Pe Number. While in natural geothermal systems, Pe numbers are between 10000-100000. To support this conclusion, a numerical study was made in the simulator TOUGH2. The model considered homogeneous and heterogeneous porous media, different permeability distributions, and two different discretization schemes to account for the grid orientation. MVF occurred at low injection rates confirming the findings of the linear stability analysis. The permeability distribution accelerated the fingers' growth and affected the thermal front, making it even diffusive and complicated. From these results, it was concluded that MVF growth dynamics could be dramatically higher in natural geothermal systems than simulated ones.

Guan characterized MVF dynamics and finger patterns at different Ca numbers using a Hele-Shaw setup [71]. In this study, a computational and experimental approach was used, having fair agreement even with reported finger patterns from the literature. Additionally, this study addressed miscible and immiscible displacements. The numerical model was based on a volume tracking formulation to describe the interface, and the author proposed a modified expression for further accuracy on the modeling of complex interfaces in the Hele-Shaw flow. The model described the growth dynamics of miscible and immiscible VF, resembling experimental results and natural morphologies. In this regard, morphology diagrams were developed for miscible and immiscible displacements for a wide range of viscosity ratios and Ca numbers at a constant flow rate. Morphology regions such as Saffman-Taylor, dendritic, coalescent, and multiscale fingers were identified. Finally, these morphologies were classified into four zones: Stable, coalescence, transition, and multiscale zones.

Ghesmat researched to determine the influence of isotropic and anisotropic dispersion tensors in the dynamics of MVF [72]. This study was conducted using linear stability analysis and non-linear simulations. It was found that isotropy in the dispersion generated finger structures like those usually found in the pure diffusive flow. On the contrary, the anisotropy altered the dynamics of fingers' growth and the interaction between them. Additionally, the author made an extensive analysis of the incidence of the velocity field to explain the new fingering dynamics and the solid dispersive effects for anisotropic systems. It was determined that broader and less complex fingers arise from strong transverse dispersive systems.

Sesini also studied the effects of anisotropy using linear and radial Hele-Shaw setups considering high mobility ratios, up to 106 [73]. The model used by Sesini was based on the mathematical formulation developed by Coutinho [63]. Unstructured grids were used under a new stabilized FEM, which improved the modeling of high mobility ratios. Anisotropic dispersion and monotonic viscosity profiles were also considered. On the one hand, the model was capable of simulating more stable sharp fronts, improving the quality of the fingers. On the other, it also identified new fingering mechanisms: Double coalescence, side branching gradual coalescence, single-sided tip-splitting, trailing lobe detachment, and alternating side branching.

The implementation of different and new numerical techniques to improve MVF modeling has also been addressed. For example, Li implemented advanced numerical techniques to simulate MVF in *2D* and *3D* [74]. The developed model was based on the Discontinuous Galerkin method with high order schemes and different structured mesh orientations to reduce numerical diffusion. The model was compared to the Finite Volume Method (FVM) and linear stability analysis. The developed model described displacements in corefloods, radial displacements, and density-driven flows in heterogeneous porous media. It was found that the Discontinuous Galerkin method allowed to achieve higher computational efficiency and a better description of the fingering dynamics. It also helped reduce the numerical diffusion caused by the mesh orientation in both *2D* and *3D* simulations. On the other hand, it was found that the FVM suffered severely of mesh orientation, predicting a different behavior of MVF dynamics depending on the flow direction and face orthogonality. This condition was more evident on radial displacements, where several differences between the FVM and the Discontinuous Galerkin method were found. Finally, the Galerkin model showed a 4% difference in the growth rate compared to the linear stability analysis.

Becker also conducted a numerical study using the Galerkin method [75]. The purpose was to improve the numerical techniques applied to the Galerkin method to speed up simulations of MVF at a large scale in *2D* and *3D*. For this purpose, it was proposed two different alternatives: i) a p-adaptive scheme, and ii) using an Algebraic Multi-Grid preconditioner on the solver. The first technique was focused on solving the fingertips where the concentration gradient was the largest. In comparison, the second technique speeded up the solver. It was found that these combined techniques allowed to simulate miscible MVF in *2D* and *3D* at high Pe numbers and mobility ratios. These techniques helped avoid using high-order schemes and refined meshes to simulate MVF even at high Pe numbers.

MVF involving three-phase flows was studied by Moortgat [76] for lateral displacements, focusing on the effects of gravity. Moortgat studied MVF for *CO*_*2*_*/methane* (80/20*%mol*) injections, compositional oil displacements, and Water Alternating Gas schemes in *2D* and *2D*. This study analyzed: Fickian diffusion, mechanical dispersion, the influence of different flow rates, different geometrical domains, heterogeneous porous media, gravity, and several relative permeabilities. The numerical model was based on the conservation equation, Darcy's equation, and a pressure equation for the compressible flow.

The model could describe MVF for compositional displacements. Also, displacing and displaced fluids of equal density were considered with the purpose of avoiding gravity override. Nevertheless, it was found that gravity had a significant effect on lateral displacement. From one side, the displacing gas tends to go downwards, displacing at the bottom of the reservoir. Local variations of densities caused this due to the evaporation of methane from the oil. Another effect of this behavior was tiny gravity fingers going upward the oil phase while the main gravity finger went through the bottom. Finally, an analysis of using or not gravity was made, concluding that accounting for the gravity effects caused an early breakthrough, around 22% earlier. Moortgat also studied the effects of considering mechanical and Fickian dispersion on MVF simulations, which only contributed to the elongation of the finger pattern. Also, considering diffusion and dispersion slightly changed the fingering pattern in the gravity and no-gravity simulations. However, it did not change the production rates or breakthrough times significantly.

Moorgat determined that gravity competes with the viscous flow only at low flow rates regarding the influence of the gas injection rate. Diffusion, capillarity, and gravity dominated the displacement. The analysis on the effects of the mobility ratio on MVF showed that for compositional multiphase flows at mobility ratios below *0.1*, the displacement front was stable. This was caused by an increase in the viscosity of the gas behind the front. However, at mobility ratios above one and increasing relative permeabilities of gas, gravitational fingers appeared. Regarding the Water Alternating Gas scheme, the model predicted that gas fingered very rapidly through the water. Also, at high permeabilities, segregation of the phases was favored. Therefore, it was found that water tends to go to the bottom while the gas is segregated at the bottom. Moreover, it was determined that Gas Alternating schemes did not increase the sweep efficiency than gas injection. Finally, for the *3D* numerical study, which was made only for gas displacements, it was found that at low vertical permeability and neglecting the gravity, the breakthrough time and oil recovery were the same compared to the *2D* case. On the contrary, when gravity was considered, the breakthrough times were delayed compared to the *2D* model.

An analysis of the effects of large aspect ratios, viscosity ratios, and transverse diffusivity was made by Hamid [77]. Primarily to determine the behavior of MVF on large aspect ratios. This numerical study was considered five different aspect ratios and four different mobility ratios, up to 30 and 100, respectively. It was found that despite many fingers generated at the beginning of the displacement, mainly one or two dominant fingers prevailed at late stages. Even more, fingers coalesce and thicken into one at high transverse diffusion numbers (>0.02). Finally, the model was found to better predict oil recovery and breakthrough times than the Todd-Longstaff empirical model compared to experimental data for different transverse diffusion numbers. This last result suggested that empirical models should not be used along calibrated reservoir simulations to estimate field-scale performance.

With the progressive improvements in computational power, more complex and robust numerical models have been developed to study MVF. Nijjer conducted a study of high-resolution numerical simulations to study the life cycle of miscible MVF [78]. The simulations were based on a sixth-order FDM along with a third-order Runge-Kutta solver. In this study, it was determined that the life cycle of MVF was composed of three regimes: i) a linearly unstable regime, ii) nonlinear finger interactions, and iii) a single finger flow. Also, the numerical simulations allow determining that the first regime was driven by advection and diffusion, independently of the width of the porous media. For the second regime, the fingering was driven by large viscosity and velocity gradients and small diffusivity. Finally, the third regime appeared when the instability diffused transversely along the width of the domain. The numerical experiments also allow determining that at small Pe numbers, the instability tends to be suppressed. Therefore, it was determined a critical Pe number where the flow tends to remain stable. Moreover, an analytical model was proposed for the second regime to calculate the transversely averaged concentrations, showing fair agreement with the numerical model and other empirical models.

### 5.2 Modeling of immiscible flows

Claridge made one of the very earliest works in 1983 on the modeling of *CO*_*2*_ stimulation [79]. This numerical study focused on the implementation of a model capable of describing IVF in heterogeneous formations during *CO*_*2*_*/steam* injection. The simulator used was a code provided by Todd, Dietrich & Chase Inc, and it was applied to a field case in the Cold Lake Upper McMurray Formation in Canada. The heterogeneity of the field was accounted for by considering 40 layers of the reservoir formation. It was found that the code could describe and reproduce the unstable displacement of *CO*_*2*_*/steam* injection. Moreover, representation of IVF was achieved at mesoscale with tongue like shapes at the displacement front. Nevertheless, the author recognized some limitations on the model as it could not reproduce severe fingering in some expected regions, despite having accounted for field heterogeneity and having used a refined mesh.

Tang studied the fractal nature of IVF in *2D* [58]. A computational and experimental study was conducted using five different fluids and a radial Hele-Shaw set-up. The numerical model was based on the DLA model and implemented a stochastic approach that considered the variability in the sticking probability function. This allowed considering variables such as interfacial tension, the pressure gradient, and the curvature of the interface. The model was capable of reproducing IVF and its fractal nature and described the behavior of self-similarity, meaning that the fractal number in the interface or local regions remained the same.

Zhang studied fractal IVF dynamics based on the DLA model [80]. In Zhang's work, fractured networks were studied in *2D*. Also, numerical experiments were conducted for radial displacements. It was found that the fractured geometry had a strong influence on the displacements controlling the fingering pattern. The displacing fluid moved along the fracture, where IVF developed faster along the direction of the fracture. Therefore, it was determined that the sweep efficiency along the fracture was higher than the areal sweep efficiency, especially if the fracture intersected the wellbore. On the contrary, areal sweep efficiency increased while sweep efficiency decreased when the wellbore did not intersect the fracture. Lastly, the author suggested handling production schemes in fields with fractures to maximize recovery efficiency.

**Figure 13:**
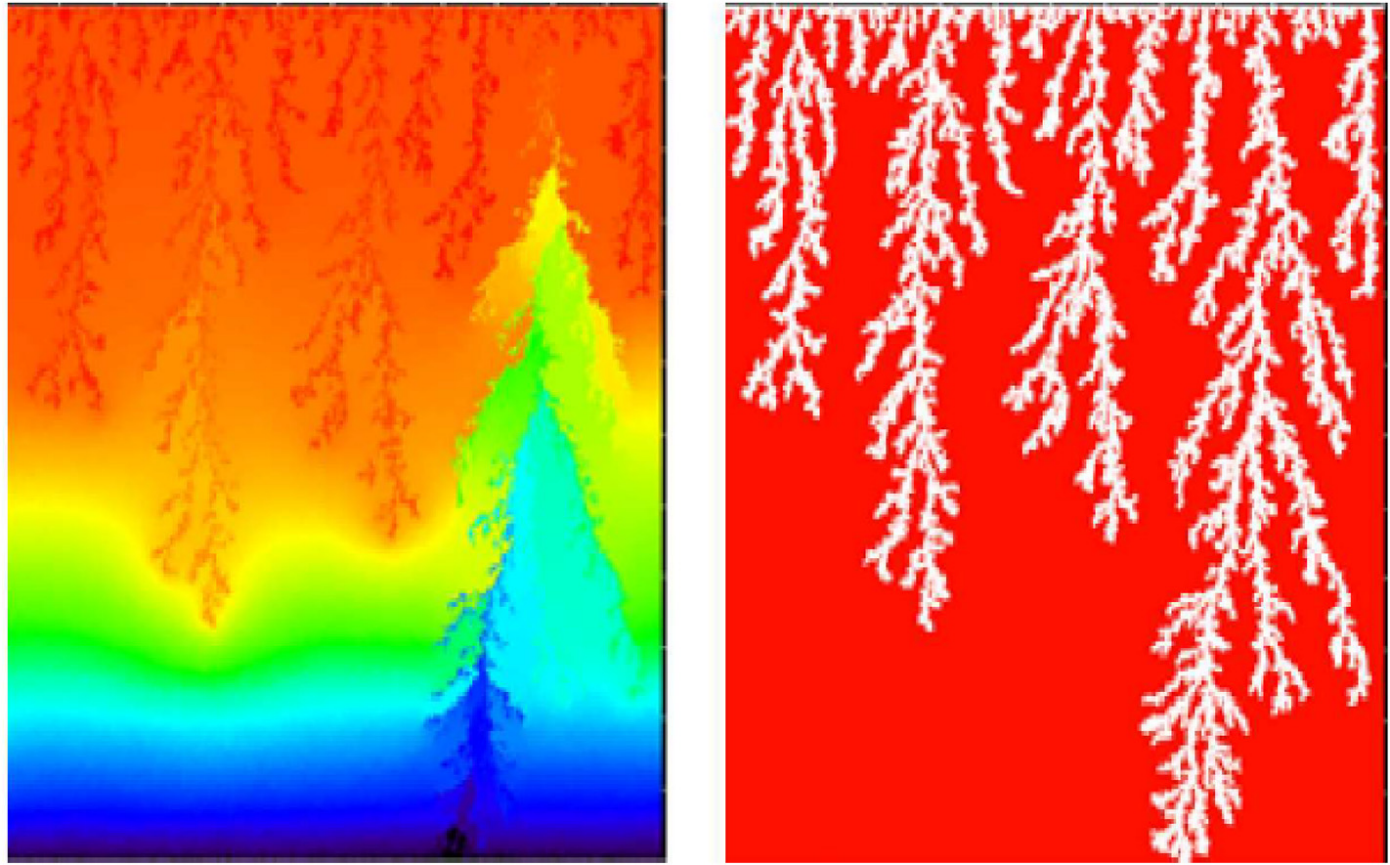
VF and its fractal nature using the Network Model. Pressure field during VF adapted from Regaige [82].

Medici also conducted a study on the fractal nature of IVF, however, not based on the traditionally used DLA method. Instead, Medici conducted research using the Network Model [81]. The influence of different aspect ratios and injection rates on IVF was studied, considered a viscosity ratio of 0.001. It was found that the Network Model could reproduce not only IVF but also its fractal nature. Additionally, it was determined that the aspect ratio increased in the flow direction, in this case, the vertical direction, but the pressure drop through time also increased in the same proportion. Finally, it was found that an increase in the aspect ratio in the horizontal direction caused a decrease in the pressure drop. An example of fractal IVF using this approach is presented in Figure 13.

Further study of VF based on Network Models was made by Regaige at length scales up to *30 × 30cm* in *2D* and *3D* for linear displacements [82]. The purpose was to study the thickening of the fingers in extra-heavy oil displacements after a breakthrough. The developed numerical model was based on a Poiseulle-type solution and was validated against experimental data found in literature and from experimental displacements in sandstone slabs of *30 × 30cm*. It was found that the model could describe IVF at a wide range of viscosity ratios for gas and liquid displacements. For the comprehension of the finger thickening, simulations of waterflooding for two different extra-heavy oils were considered. It was found that a noticeable change caused the thickening of the pressure drop along with the porous system after the breakthrough. With no significant pressure gradient, capillary forces became more substantial than the viscous ones, causing the fingers' thickening in pores with low capillary resistance. The author also made a sensibility analysis on the influence of domain size, wettability, and injection rate on the thickening of fingers. It was found that more considerable lengths, higher injection rates, and displacing fluid-wet systems favored the thickening of the fingers, improving sweep efficiency.

The Fractal VF studies reviewed have been conducted for *2D* systems for both miscible and immiscible displacements. Tian conducted a *3D* study of IVF based on the DLA method on real rocks/cores [83]. The porous media was obtained by scanning and digitalizing cores. Moreover, the digital cores were subjected to a processing technique called Random Motion of Heuristic Particles to determine the connectivity of the pores. The displacements were made using oil and *CO*_*2*_ for several viscosity ratios and injection rates. It was found that the *3D* model could describe a decrease in displacement efficiency, and fractal dimension, for increasing mobility ratios. Finally, the model could describe the fingering dynamics qualitatively in *3D* in real porous media.

A study that used the governing equations of fluid dynamics to simulate IVF was made by Nakayama in *2D* [84] for gas-liquid displacements in a Hele-Shaw cell. The model could accurately solve the velocity and pressure field accurately and reproduce IVF growth dynamics. Additionally, the interface tracking method was achieved by considering the interface as a density discontinuity zone. Finally, the model described characteristic dynamics of IVF, such as finger birth, split, and shielding.

Modeling of VF in *3D* is computationally more expensive and complicated than its *2D* counterpart. Belotserkovskaya [85] developed a numerical model capable of simulating IVF in *3D*. The model was based on the assumptions that the displacement had zero hydrodynamic dispersion and high Ca numbers. Additionally, the model was based on a high-order accurate finite-volume weighted non-oscillatory scheme, capable of emulating IVF dynamics in *3D*. It was found that this high-order approach was capable of successfully emulating IVF dynamics and could determine that tip splitting was the primary or dominant pattern during the displacement.

Another approach to model VF based on fluid flow governing is using the Lattice-Boltzmann Method (LBM). Dong used this approach to study the effects of Ca number, Bond (Bo) number, viscosity ratio, and wettability at mesoscale [86, 87]. In Dong's first study, five different mobility ratios were considered in a *2D* flow in a channel [86]. It was found that IVF could be modeled using the LBM, where the model could predict and emulate the fingering growth dynamics at low and high Ca numbers. Also, it was found that using this numerical approach, the finger length did not increase with the viscosity ratio at constant injection rates. However, under non-wetting displacing conditions, the finger growth was found to be faster. On the contrary, displacing wetting conditions, the displacement was more stable, taking longer to achieve a breakthrough. Finally, this work, and Dong's second study, made an extensive analysis of gravity's influence, concluding that this variable should always be considered as it significantly affects the fingering growth dynamics.

On the other hand, Dong's second study analyzed the effects of Ca and Bo number, viscosity ratio, and wettability on IVF. For this purpose, a Hele-Shaw setup and a simplified porous media were considered to validate the numerical model [87]. The computational results showed fair agreement with the Hele-Shaw experiments, especially at emulating IVF growth dynamics and predicting an accurate areal sweep efficiency. Moreover, the numerical model could determine wettability properties such as contact angle and solid-fluid strength, which affects the dynamics of IVF. Lastly, it was demonstrated that wetting displacing fluids reduced IVF, achieving higher sweep efficiencies in both the Heel-Shaw cell and the simplified porous media.

**Figure 14:**
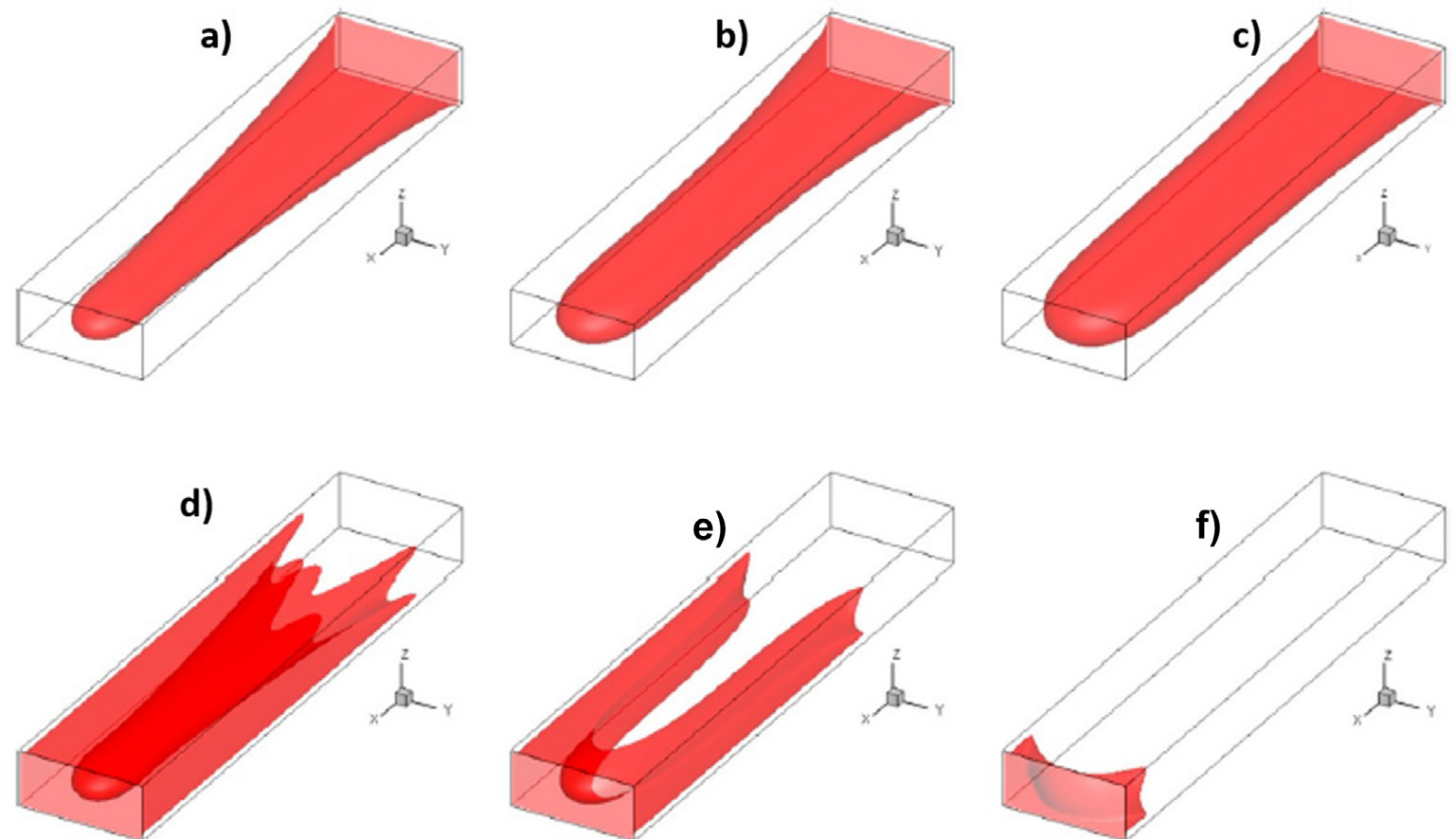
Results of the LBM prediction of VF at a Ca number of a) 0.1875, b) 0.0313, c) 0.0078 and a contact angle of 180°, and a Ca number of d) 0.1875, e) 0.0313, f) 0.0078 and a contact angle of 90°. Image adapted from Shi [88].

Newtonian and non-Newtonian displacements in *2D* and *3D* were studied using the LBM by Shi [88]. The study of Shi focused on analyzing the effects of the Ca number, viscosity ratio, wettability, gravity, system dimension (*2D* vs. *3D*) and rheology on IVF in a channel. It was found that during the immiscible displacements in a channel prevails the formation of one dominant finger. Moreover, it was found that the dynamics of the finger growth in *2D* and *3D* were utterly different. First, in *3D* the finger width growth was influenced by the spatial dimension and the wettability with the surrounding walls. Also, the results showed that at increasing Ca numbers and viscosity ratios, the fingers tend to become significantly narrower, causing earlier breakthrough times. On the other hand, it was possible to determine that the gravity effects caused that the fingers grew asymmetrically. Lastly, the rheological behavior of the fluids noticeable influenced the fingering pattern. It was determined that shear-thinning fluids tend to cause more stable displacements than the shear-thickening ones, reducing IVF. Shi's results are presented in Figure 14 for different wettability conditions and Ca numbers.

Further study of non-Newtonian VF using the LBM was done by Wang [89]. In this study, the displaced fluid was a non-Newtonian shear-thinning fluid with Power-Law viscosity. It was found that a heterogeneous viscosity field dominated IVF. It was identified that the strong shear-thinning behavior caused weaker interfacial instabilities and thicker fingers, achieving better displacement efficiency. Also, the heterogeneous viscosity field was favored by the low viscosity region of the non-Newtonian fluid affecting the finger front. Lastly, the Effective Field Viscosity model was developed, which quantified the heterogeneity of the viscosity field to determine effective mobility ratios using these kinds of fluids.

Shiri analyzed the influence of five different flow rates, viscosity ratios, and wettability on the dynamics of IVF in microfractures and porous media [90]. In this study, the LBM was used based on the Shan-Chen formulation. Regarding the influence of the flow rate, it was found that the displacement front in the fracture and the porous media tend to be stable and piston-shaped at low flow rates. On the other hand, at high flow rates, IVF was dominant in the porous media, while a single wide finger was dominant in the microfracture. However, it must be mentioned that this study was carried at viscosity ratios below one. The author mentioned that the Shan-Chen model failed at viscosity ratios above five. Even so, the results showed that IVF occurred in the porous media at viscosity ratios below one. Lastly, it was found that systems with a contact angle between 90 and 150° had low sweep efficiency.

Polymer flooding has also been studied under the LBM at pore scale by Wei, considering three phases: water, oil, and polymer [91]. In its *2D* LBM model, the amphiphilic structure of the surfactant was considered to account the microscopic fluid interactions. To achieve this, the LBM was based on a Boltzmann-BGK formulation coupled with the two-dimensional nine-velocity model and the Shan-Chen multiphase model. The advantage of this sophisticated LBM was that the surfactant could be modeled at a kinetic level, accounting for the particles' distances and dipolar orientations, giving more fidelity to the displacement process on the polymer flooding modeling. Therefore, the model could predict the reduction of interfacial tension, emulsification and wettability alteration. With these capabilities, the model could predict an increase in sweep efficiency caused by a dominant CF and attributed to reducing the interfacial tension. Also, emulsification and wettability alteration prediction allows modeling Oil-in-Water microemulsions and the subsequent phase distribution within the porous system. Lastly, it was found that at different wettability conditions, there was remaining oil in the porous media which was called “hidden oil on the down-gradient side of the walls.” This study suggested that this remaining oil could be recovered by changing the direction of the flow.

LBM has also been used to study *CO*_*2*_ geosequestration in aquifers by Yamabe [92]. This study was made at pore scale in *3D*, focusing on the effects of Viscous and Capillary Fingering on the *CO*_*2*_ geosequestration process. Moreover, the model could capture a phenomenon called Haines jumps which is a condition of burst flow during the CF regime. This study also considered different injection rates and pressure gradients to control the displacement mechanism (Viscous or Capillary Fingering). It was found that during *CO*_*2*_*/water* displacements, two kinds of Haines jumps can occur: Forward and backward, both affecting differently the *CO*_*2*_ saturation during the displacement. For example, it was determined that *CO*_*2*_ saturation increased with backward Haines jumps, while forward jumps caused the contrary effect.

**Figure 15:**
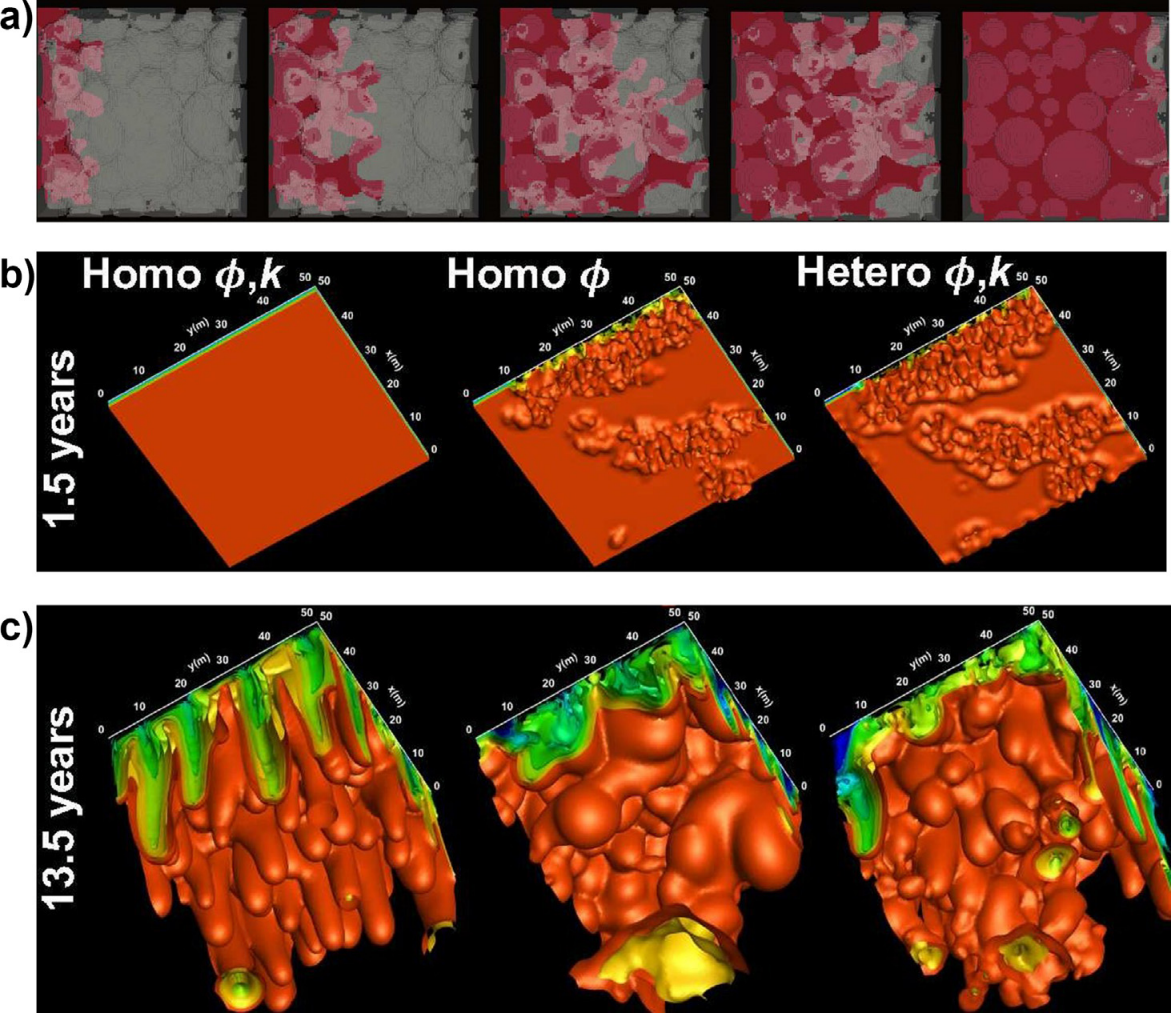
VF in *CO*_*2*_ sequestration at a) Pore-scale at different times, adapted from Yamabe [92]. b) Mesoscale accounting field heterogeneities, adapted from Soltanian [93].

Another researcher that studied IVF dynamics of *CO*_*2*_ sequestration was Soltanian who conducted a *3D* study to determine the impacts of facies connectivity during the displacement and mixing of *CO*_*2*_ [93]. In this case, the discontinuous Galerkin method was used to develop high-resolution numerical simulations of *CO*_*2*_ displacements in heterogeneous media. The heterogeneous porous media was developed based on two models: i) a binary heterogeneity model and ii) a multiscale heterogeneity model. These models were used to represent the deposition architectures or geometrical structures. As a result, the domain developed consisted of a heterogeneous porous media of *100 × 100 × 15m*. The results showed that the model could describe the advection-diffusion transport of *CO*_*2*_ in heterogeneous porous media. Also, it could describe the effects of homogeneous and heterogeneous porous media in terms of *CO*_*2*_ saturation through time. It was found that *CO*_*2*_ sequestration can be divided into four flow regimes: i) Diffusive transport, ii) Fingering and convection-dominated transport, iii) Fingering stagnation, and iv) advection dominant transport. Moreover, these flow regimes appeared on homogeneous and heterogeneous media. Some examples of *CO*_*2*_ IVF results are presented in Figure 15.

Studies on numerical techniques to improve predictions about IVF Dynamics on numerical simulations were made by Mostaghimi [94, 95]. Using adaptive mesh techniques to improve the numerical modeling of IVF based on the FVM using unstructured meshes was proposed. The purpose was to correct the issues of numerical dispersion caused by the structured grids commonly implemented on reservoir simulators. The model could refine the mesh in evolving flows at the interface of the fluids to enhance the modeling of fingering dynamics. In contrast, coarsened the mesh in bulk regions of both phases, balancing the computational cost and reducing numerical dispersion. It was found that the model could describe with higher accuracy saturation profiles compared to commercial simulators, in this case, the code Eclipse 100 using high-resolution meshes. Moreover, the adaptive mesh technique had a similar computational cost compared to the finer meshes used in commercial software. Nevertheless, despite it was found that the meshes used in the commercial software suffered from numerical dispersion, this issue only affected the fingering pattern. As a result, the adaptive mesh model and the commercial software had similar predicting breakthrough and water-cut profiles.

**Figure 16:**
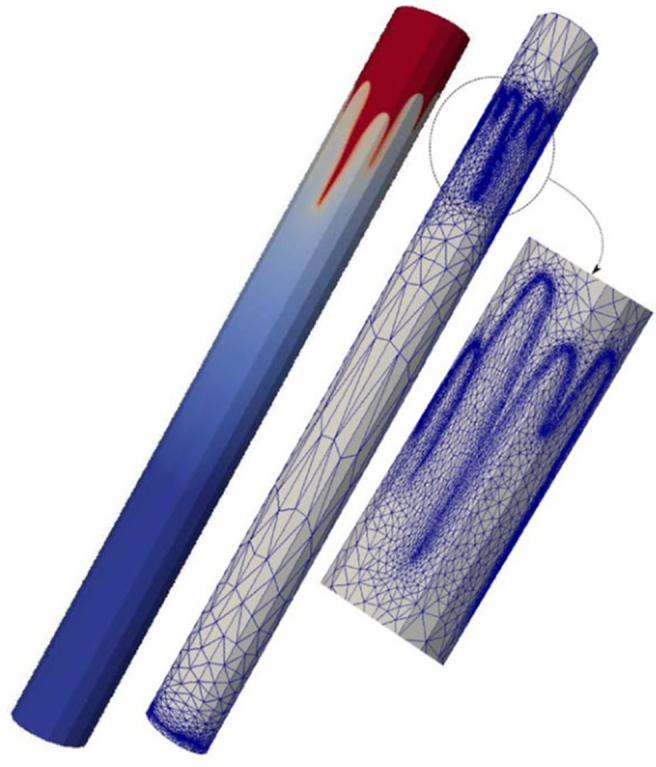
Adaptive mesh example in *3D*. Image adapted from Kampitsis [99].

Further studies on improving meshing techniques for the modeling of IVF were made by Adam in *2D* and *3D* using the reservoir software IC-FERST [96]. The study consisted of comparing structured and unstructured meshes to determine which one is more suitable for the modeling of IVF. First, it was found that the unstructured mesh did not suffer from numerical diffusion, as happened with its structured counterpart. On the contrary, this last one predicted different breakthrough times on numerical replicas showing oscillatory convergence. Therefore, it was suggested the usage of unstructured meshes for the modeling of IVF. Regarding the adaptive mesh, it was found that this technique reduced the computational time compared to fixed meshes up to 10 times, as they can save up to five times the number of elements required on the mesh. Finally, this mesh technique allowed to keep consistency on the prediction of breakthrough times and the description of the fingering dynamics. An illustrative example of the adaptive mesh technique is shown in Figure 16.

Displacements considering three-phase flow were studied by Lagreé [97]. Numerical simulations using the software GERRIS were conducted to study the displacement of two subsequent injections with different fluids. This to emulate two different recovery techniques on homogeneous and heterogeneous porous media. The numerical model was based on the Volume of Fluid (VOF) method and solved in GERRIS. Additionally, an adaptive meshing technique was implemented to improve the modeling of the three-phase IVF. First, it was studied a two-phase flow involving a single injection. Here, it was found that the model could accurately predict IVF growth dynamics emulating Saffman-Taylor like fingers. Moreover, the results indicated the VOF method could predict a fractal dimension of 1.74, which was close to the one found by the DLA method, 1.713. Also, the pressure field was measured before and after the breakthrough, finding that the pressure difference along the domain did not drop dramatically at the breakthrough. Instead, with this low change, the overall production rate driven by the pressure gradient remained close before and after the breakthrough. Regarding the two-injection three-phase flow analysis, the injection scheme was as follows: first, it was injected a less viscous fluid into the domain, and just before the breakthrough, was injected an intermediate viscosity fluid. In this case, it was observed that injecting an intermediate viscous fluid led to a higher sweep efficiency, confirmed by experimental results.

## 6 Modeling through reservoir simulators and CFD

So far, extensive research has been developed to understand VF, as shown in the previous sections. Miscible and immiscible displacements have been studied for several viscosity ratios, different porosities, and permeabilities for homogeneous and heterogeneous media and different fluids, including gases and liquids with different rheological behaviors. Derived from these studies, several models and correlations have been created to predict and emulate the behavior of displacements accounting for the effects of VF. Some of these models have been implemented in Reservoir Simulators' commercial codes to improve the accuracy of the modeling of oil recovery. These works will be reviewed in Section 6.1.

On the other hand, as reviewed in Section 5, there are several numerical approaches to model VF, especially at the microscale. Diffusion-Limited Aggregation, Galerkin, LBM, Direct Numerical Simulation (DNS), and CFD have been the main approaches to simulate VF. However, each approach has its advantages and disadvantages, such as the substantial computational cost, the limitation of modeling up to a certain length and time scale, the capability to model the phenomena, including fractal behavior, etc.

Section 6.1 will review VF effects in Reservoir Simulation codes and the advancements in modeling this phenomenon. This powerful approach has been the tool of choice for the advanced modeling of oil-water flows in oil reservoirs at macro and mesoscale. Nevertheless, CFD has arisen as a new technology to model oil displacements. Even more, it has shown evidence to model VF explicitly based on the solution of the governing equations of fluid flow. Section 6.2 will briefly explain this computational technology, its shortcomings, and the main contributions to the modeling and understanding of VF. Also, in Tables 5 and 6, the main data of the numerical experiments used in these studies are summarized.

**Table 5:** Summary of the numerical setups and fluids used for the modelling of miscible displacements using the reservoir and CFD simulators.

Author	Model/Code used	Fluids	Density $\left(\frac{kg}{m^{3}}\right)$	Viscosity (*P a s*)	Porosity	Permeability (*m*^*2*^)
Sorbie 1991 [100]	Direct Numerical Simulation/In-House	- 3 Brines - 3 Glycerol solutions	*1st brine*: 1138 *2nd brine*: 1193 *3rd brine*: 1222 *1st glycerol*: 1136 *2nd glycerol*: 1195 *3rd glycerol*: 1224	*1st brine*: 1.55 × 10^−3^ *2nd brine*: 1.9 × 10^−3^ *3rd brine*: 2.1 × 10^−3^ *1st glycerol*: 5.58 × 10^−3^ *2nd glycerol*: 22.69 × 1^−3^ *3rd glycerol*: 64.74 × 10^−3^	0.38	6.41–7.4 × 10^−12^
Christie 1993 [101]	Finite Volume/In-House	- Oil - Water - Gas	674.37 999.55 384.44	1.3 × 10^−3^ 0.31 × 10^−3^ 4.5 × 10^−5^	0.2	1.97 × 10^−13^
Taheri 2014 [102]	COMSOL/Commercial	- Oil - Solvent	NR	RTM	0.312–0.476 (RTM)	532.7–3650 (RTM)

**Table 6:** Summary of the numerical setups and fluids used for the modelling of immiscible displacements using the reservoir and CFD simulators.

Author	Code used	Fluids	Density $\left(\frac{kg}{m^{3}}\right)$	Viscosity (*P a s*)	Interfacial tension (*N/m*)	Porosity	Permeability (*m*^*2*^)
Rubin 1993 [103]	VIP (Nexus)/Commercial	- Oil - Gas	RTM	1.03 × 10^−3^ 1.55 × 10^−5^	NR	NR	RTM
Godderij 1995 [104]	Probabilistic-Finite Volume/In-House	- Oil - Steam	NR	RTM	NR	NR	RTM
Verga 2007 [105]	GepQuest/Commercial	- Oil - Water	876 1060	5 × 10^−4^ -0.05 (RTM) 0.001	NR	0.2	4.93 × 10^−14^- 9.86 × 10^−13^ (RTM)
Makinde 2011 [106]	Eclipse 100/Commercial	- Oil - Gas - Water	384.44 0.657 1050	RTM 5 × 10^−4^ NR	NR	0.156	1.97 × 10^−13^-3.94 × 10^−12^ (RTM)
Yue 2012 [107], 2015 [108]	Finite Volume/NR	- Oil - Water	850 1050	15 × 10^−3^ 1	NR	NR	1.54 × 10^−7^
Akangbou 2017 [109]	Finite Volume/NR	- Oil - Water	929.07 1028.38	3.8 × 10^−3^ 48 × 10^−4^	NR	0.35	6.90 × 10^−12^
Luo 2016 [110], 2017 [111], 2017 [112], 2017 [113]	UTCHEM/Commercial	- 5 Oils - Water - Polymer	NR	*1st oil*: 0.06 *2nd oil*: 0.56 *3rd oil*: 1.44 *4th oil*: 5.2 *5th oil*: 5.2 *Water*: 0.001 *Polymer*: RTM	NR	0.29 (RTM for each article)	5.92 × 10^−12^ (RTM for each article)
Cuevas 2014 [114]	CFX/Commercial	- Oil - Water	897.3 NR	0.00283 2.91 × 10^−4^	0.006	0.2654	4.99 × 10^−9^
Zhu 2017 [115]	COMSOL/Commercial	- Oil - *CO*_*2*_	100 1000	1 10	24.5	RTM	RTM
Gonzalez 2014 [116]	FLUENT/Commercial	- Oil - Water	973.2 998.2	0.037 0.001	0.517	0.28	RTM
Wijeratne 2015 [117]	FLUENT/Commercial	- Silicone Oil - Crude Oil - Water - Resin	970/920 920 1000 1200	0.45/0.15 0.3 5 × 10^−4^ 0.066	*Corefloods*: 0.0075 *Reservoir*: 0.0025	*Corefloods:* 0.38 *Reservoir:* 0.3	*Corefloods:* 1.77 × 10^−12^/6.9 × 10^−13^ *Reservoir:* 2.96 ^×^ 10^−12^
Wijeratne 2015 [118]	FLUENT/Commercial	- Oil - Water	920 1000	0.3 5 × 10^−4^	0.0025	0.3	2.96 × 10^−12^
Ahmadi 2018 [119]	COMSOL/Commercial	- Oil - Water	948 988	0.096 0.001	0.032	NR	RTM

### 6.1 Modeling of VF through reservoir simulators

Sorbie conducted one of the first studies comparing the accuracy between empirical models (derived from the fractional flow theory) and numerical codes [100]. In this study, three models were compared against experimental data and DNS. The empirical models tested were: Koval [120], Todd and Longstaff [121], Fayers [122], and Odeh and Cohen [123]. This study allows determining which model could be used in multidimensional simulations, along with their respective drawbacks. It was concluded that Todd and Longstaff's model was the most reliable as it could reproduce the experimental pressure drop and the effluent profile. However, it was also found that the DNS reproduced better the experiments outperforming the empirical models.

Rubin implemented a compositional VF correlation in the commercial code VIP (Nowadays Nexus) to enhance unstable gas displacements in *1D* [103]. Additionally, the modified code was also capable of simulating immiscible displacements. The implemented model was compared to compositional *2D* simulations. Computational experiments of different gas injections were conducted: Immiscible gas, vaporizing gas, condensing gas, and condensing/vaporizing gas was studied. It was found that both, the implemented model in the reservoir code and the compositional simulations, were capable of simulating VF sharing the same results.

Computational studies of Water Alternating Gas schemes in *3D* were conducted by Christie [101] to recalibrate the Todd and Longstaff model for buoyancy and gravity-driven miscible displacements. It was used as an in-house code [124], and it was compared against DNS for validation. It was found that the mixing parameter of the Todd and Longstaff model should be recalibrated, whereas the models' accounts or not the gravity body force. Therefore, the author suggested a methodology or procedure to recalibrate this parameter for both kinds of displacements (gravity and buoyancy-driven). As a result, with the procedure for the recalibration of the mixing parameter it was possible to avoid high-resolution simulations, especially to determine this parameter.

Godderij made further studies to improve gas injection modeling to predict and describe steam displacements accounting for VF and steam override [104]. The pseudo-*3D* model accounted for gravity, viscous forces, heat and mass transfer, and heterogeneous porous media. Additionally, the model was tested in simulations of a quarter five-spot pattern for a medium viscosity oil. It was found that the developed model could describe steam displacements, including VF and channelings, without the necessity of fine grids, especially for heterogeneous systems. Also, the results showed that heat losses had a stabilizing effect suppressing VF, which was not decisive for the medium viscosity oil and the consequent steam override. On the other hand, the results on the homogeneous porous media showed that VF and steam override were more severe, having lower recovery efficiency.

A study that questioned and analyzed the advantages and disadvantages between analytical and numerical models for predicting water conning and cresting was made by Verga [105]. This study compared seven empirical correlations for vertical wells (Water Conning) and 3 for horizontal wells (Water Cresting) against numerical simulations made in GeoQuest and field data for history match. The compared variable was the critical rate, which is the maximum rate of oil that can be produced before the water breakthrough. The numerical models were made using a hexahedral grid. The vertical well was based on a radial model, while the horizontal well was based on a *2D* cross-section model. It was found that the analytical solutions differed significantly from the numerical ones. For the water conning case, it was found that the correlations underestimated the critical rate. On the other hand, for the water cresting, the correlations overpredicted the critical rate. On the contrary, the numerical model had a lower error than the empirical correlations and the field data, suggesting its superiority, especially against horizontal well correlations—moreover, the numerical model allowed to visualize the cresting and conning phenomena along the reservoir.

Regarding the modeling of horizontal wells, Makinde conducted a study to predict the performance of the wells after breakthroughs derived from water conning/cresting, a consequence of VF [106]. The purpose of this study was to model Horizontal wells using the software Eclipse 100 in order to develop analytical correlations capable of predicting water breakthrough and post-breakthrough well performance. The results obtained with the simulator and the developed correlation were compared against other classical correlations and field data reported in the literature. It was found that the simulator and the new correlation gave an accurate prediction of the breakthrough times compared to the field data. Lastly, the numerical model allowed determining that longer perforations in the well completion could cause a reduction in the Water-Oil-Ratio (WOR) after the breakthrough.

The study of barriers along horizontal wells has also been addressed. Yue conducted a numerical study on the influence of impermeable and semi-permeable barriers to avoid water cresting [107, 108]. In this study, two barriers were considered: i) semi-permeable and ii) impermeable. For the impermeable study case [107], a series of numerical experiments were conducted to obtain an analytical model capable of predicting the critical flow rate for water cresting. It was found that the model could describe quantitatively and qualitatively not only water cresting but also the incidence of impermeable barriers. It was determined that increasing barrier sizes and distances from the well delayed the breakthrough. For the study of the semi-permeable barriers [108], similar results were found. Increasing the size and distance from the well helped to increase the critical rate for water conning. Nevertheless, it was found that for semi-permeable barriers at larger sizes and increasing permeabilities, critical flow rates and breakthrough times tend to decrease.

Akangbou also conducted a numerical study of impermeable barriers in horizontal wells [109]. In this case, the effects of the orientation of the barrier on the well performance were studied. Different horizontal and inclined barrier sizes were considered to determine which one mitigated the best water cresting. It was found that the thickness of the horizontal impermeable barrier affected oil recovery. Contrary to the results found by Yue [107], Akangbou found that despite higher thickness mitigated water cresting, it also reduced oil mobility around the barrier, affecting cumulative production.

**Figure 17:**
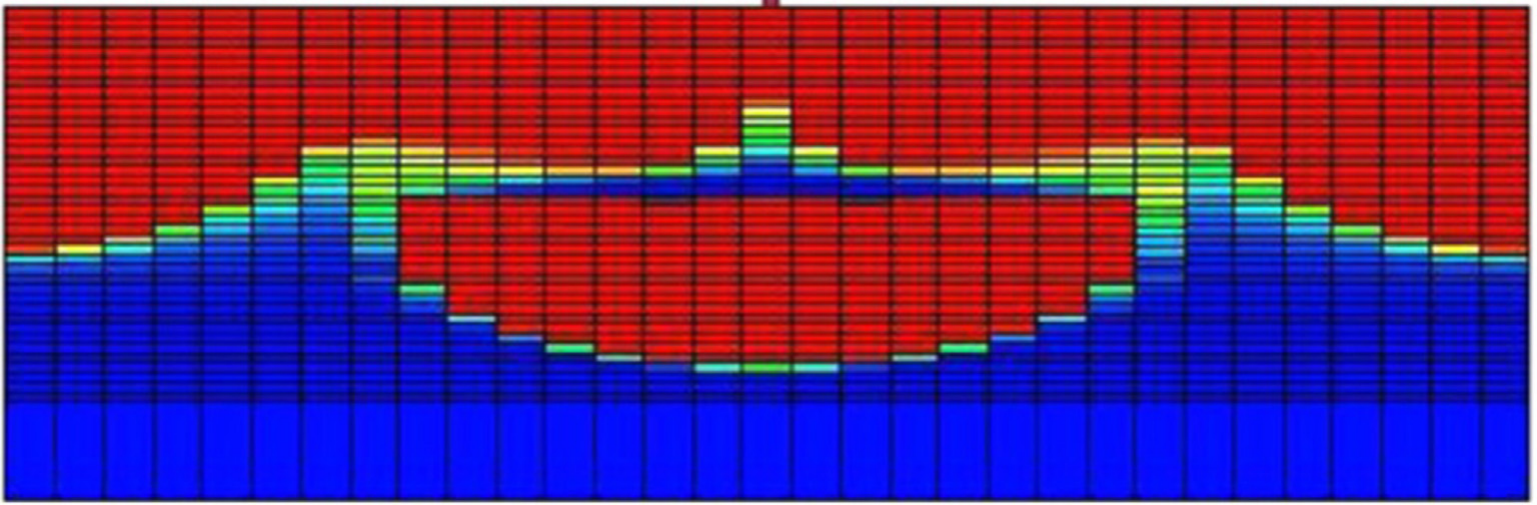
Results on the modeling of impermeable barriers on horizontal wells, adapted from Yue [108].

Additionally, it was found that horizontal barriers were more effective than inclined ones. Lastly, it was suggested that an optimum size and location could be determined to achieve maximum efficiency. Results on the modeling of VF and water cresting are presented in Figure 17.

Implementation of VF models in reservoir simulators for the accurate modeling of this phenomenon and its incidence on oil recovery was made by Luo [110, 111, 112, 113]. Luo's studies focused on several flooding techniques such as waterflooding and chemical flooding in *1D*, *2D*, and *3D* models made in the software UTCHEM. The purpose of this work was to implement VF models capable of resolving this phenomenon in the large grid sizes, which are commonly used in reservoir simulators. Moreover, to validate the model for the different EOR techniques studied, a history match was made using heavy oil field data.

Luo's First studies focused on presenting the Effective-Finger model (EFM) [110, 111]. This model was developed to predict VF in heavy oil reservoirs, and it was based on the dynamic division of the flow domain in three zones: i) water/chemical two-phase flow zone, ii) oil zone and iii) bypassed oil zone. Additionally, this model included parameters that self-adjusted at each cell to perform accurate modeling of displacements accounting VF, correlating the local finger number with the model parameters. First, it was found that this model was capable of reproducing coreflood displacements for several injection rates and fluids, matching pressure drop and oil recovery experimental data accurately. Moreover, it was found that the model was more reliable compared to other classical models, for example, the Corey Function method.

The model could reproduce VF for field cases of waterflooding and polymer flooding at a reservoir scale, accounting for heterogeneous porous media. Moreover, while the classical simulations could not reproduce the fingering phenomenon, Luo's model could reproduce VF for several injection rates on both recovery techniques. Regarding the polymer flooding, the new model could simulate the rheological shear-thinning behavior of the polymer, which resulted in a rise of bypassed oil. Quantitatively, it was found that the classical models over-predicted oil recovery while the EFM gave a more reliable prediction. Lastly, the new model allowed the coarsening of the meshes used in the simulations, reducing computational Costs while keeping reliable results converging on the exact profiles of pressure drop and oil recovery.

On the other hand, Luo's presented the results of the history match for real field scenarios on a second study, especially for the polymer flooding case [111]. This study showed the modeling of a heavy oil field composed of two injectors and three producers. History match for the produced oil and water-cut was achieved. Moreover, it was found that the implemented model had an average error of around 7%, while the classical model had an average error of around 33%. Therefore, the developed model was more reliable and computationally cheaper for reservoir simulation accounting VF.

Luo further explained the three dynamic zones concept of the EFM using water and polymer flooding examples, constantly comparing the results against conventional reservoir simulation [112]. The examples, or test cases, were made for three different length scales: Core scale (or laboratory scale), intermediate scale, and field scale. The core scale study was a comparison between the EFM and ten different coreflood experiments. It was found that the proposed model could replicate the core flood experimental results outperforming the conventional reservoir simulation, which could not match the experimental data with the same precision. Moreover, the core scale *3D* results showed that the EFM could capture the fingering patterns during the displacement, predicting cross and bypassing flows.

Regarding the intermediate scale study, the purpose was to demonstrate how the EFM can be upscaled. Also, to demonstrate that upscaling the domain size did not affect the capabilities of the new model. Therefore, in this case, a domain 6 × 105 times larger than the core scale was considered. Additionally, the grid size was about the same size as a core to illustrate that the model could capture VF using larger meshes. The upscale was made by considering the cross-sectional area changes in the “VF number,” a variable that correlated parameters that represented growth rate and strength of VF. It was found that the model was capable of representing VF at the intermediate scale and describing channelings derived from this phenomenon. Lastly, the model was applied to field-scale, considering a water and polymer flooding injection scheme. It was found that the model could predict the decadency of oil production during waterflooding and the posterior improvement in sweep efficiency and oil recovery after the polymer flooding, showing the expected profiles of oil recovery.

**Figure 18:**
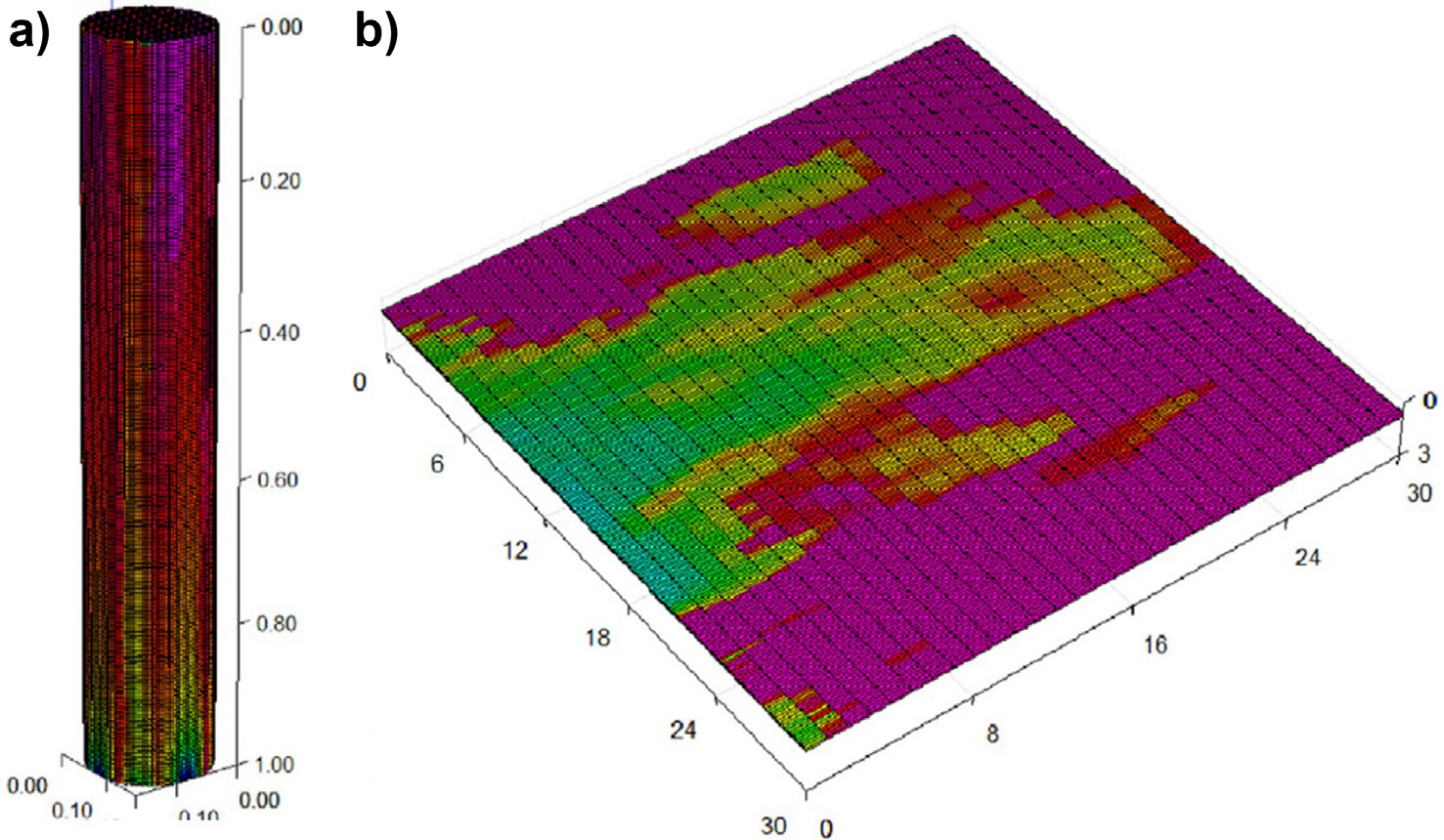
Results obtained by Luo with the Effective-Fingering Model: a) at the core scale, adapted from Luo 2018 [125]. b) at the field scale, adapted from Luo 2018 [125].

Luo's last study was about polymer flooding simulations using the EFM in layered heavy oil reservoirs [113]. Besides implementing Luo's fingering model, this study also proposed an implicit well-rate-allocation model to allocate flow rates in reservoir simulations with multiple layers properly. First, qualitatively experimental validation was made with an experimental two-layered bead-pack using Xanthan gum. It was determined that the EFM combined with the implicit well-rate-allocation model was the most appropriate way to model VF during polymer flooding in layered domains. Additionally, the combined model was extrapolated to a field-scale where a water and polymer flooding injection scheme was used. Although the classical reservoir simulation could predict channeling, Luo's model could also predict VF and enforced crossflow derived from the polymer flooding. Some of the results obtained by Luo's studies are presented in Figure 18.

### 6.2 Modeling through CFD

Relatively few studies of VF and reservoir displacements have been conducted using CFD, differentiating it from LBM and Galerkin methods which could be considered special cases of CFD. This method relies on the equations of momentum, energy, and continuity, which govern fluid flows. An advantage of this method in the study of VF is that it can represent micro, macro, and even mesoscale. This section will be reviewed the studies carried on so far regarding the modeling of VF.

At the pore scale, Taheri conducted a *2D* and *3D* study of MVF in heavy oil miscible displacements at low Pe numbers [102]. This study considered different mobility ratios, injection rates, and porous patterns to study its influence on MVF. It was found that CFD was a valuable tool to predict dilution and spreading on MVF. Additionally, the simulations allow predicting diffusion and dispersion coefficients directly from pore-scale displacements. Lastly, the model could predict the differences caused by various mobility, porous media, and Pe numbers on the fingering growth rate and mixing zone dynamics.

**Figure 19:**
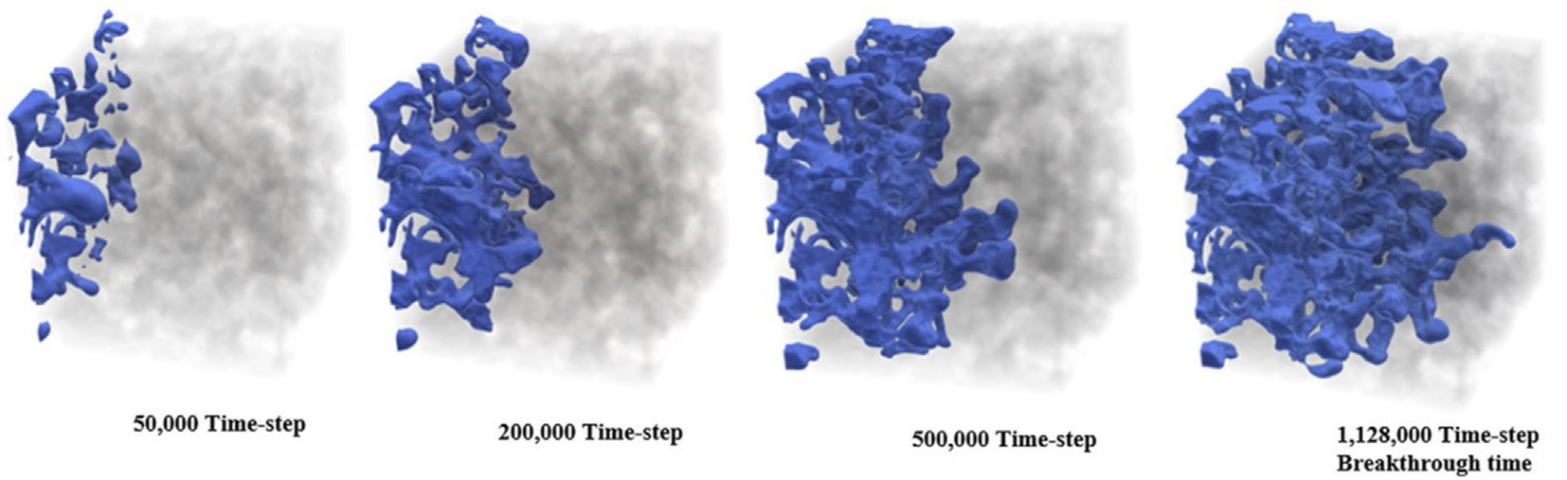
VF results using CFD codes: a) At pore scale in *3D*, adapted from Bakhshian [126].

Another *2D* study at the pore scale was conducted by Cuevas to obtain relative permeability curves for oil and water using CFD simulations [114]. In this case, the porous media was generated using an orthorhombic arrangement or distribution of equal-size spheres. The developed *2D* model was capable nof reproducing Viscous and Capillary Fingering at the pore scale, but it also proposed using a turbulence model to account for non-permanent laminar regimes led to more reliable results, at least from a numerical point of view. The model also described the pressure drop along the porous media, droplet displacements through the pores caused by capillary forces, and it was even capable of predicting relative permeability curves for water and oil. Although the model was not validated against experimental data, it was compared to analytical solutions for capillary pressure and relative permeability using the models of Young-Laplace and Honarpour, respectively. It was found that the model had fair agreement with the prediction of the capillary pressure and relative permeability curves for unsteady-state solutions, while steady-state solutions were used only to determine the absolute permeability. Lastly, the usage of the turbulence model proved to give more reliable and accurate modeling of wettability on the walls. It was found that the *k*-*ω* turbulence model with *SST* transitional model was adequate to predict not only wettability effects but also phase separation and droplet formation. Some of the results obtained through CFD modeling are presented in Figure 19.

Pore-scale *CO*_*2*_ displacements in *2D* were studied by Zhu to understand the behavior of *CO*_*2*_ sequestration in oil-wet systems [115]. It was considered a heterogeneous porous media that consisted of two sections: a small upper dense, or thicker zone and a lower zone of high permeability. Also, a wide range of capillary and gravity numbers were considered, along with different viscosity ratios, to study the *CO*_*2*_ displacements at the pore scale. It was found that gaseous displacements could be modeled with CFD emulating Viscous and Capillary Fingering. Additionally, the model also predicted the pressure profile along with the porous media through time. The simulation showed that the pressure increased while the *CO*_*2*_ front advanced, showing a peak of pressure at the finger front. Also, the model predicted the pressure drop in the system once breakthrough was achieved. The results also determine that vertical flow paths interconnecting horizontal fingers were caused by capillary and gravity forces. Nevertheless, they tend to decrease at higher flow rates. Lastly, the different wetting conditions were studied considering different contact angles, where oil recovery decreased at decreasing contact angles worsening VF.

A study to explore the possibility of use CFD as a tool to model and understand VF beyond pore-scale was conducted by Gonzalez using oil and water [116]. A *150 × 60cm* computational model was made in *2D* and validated against experimental data. It was found that the model could reproduce IVF growth dynamics, emulating: spread, split, and coalescence. Additionally, the model also calculated the velocity field, finding that the fingers travel faster than the displacing fluid. Moreover, for high viscosity oils, the model could also predict the preference of water to flow through single channels. A sensibility analysis was also made for the critical velocity and critical wavelength on parameters such as viscosities, densities, porosities, permeabilities, and flow rates. The results were compared to the Muskat model, finding that the CFD model better predicted the critical velocity than the Muskat model developed for Hele-Shaw flows.

Mesoscale computational studies of IVF for heavy oil reservoirs with aquifers were made by Wijeratne in *2D* for homogeneous reservoirs based on the VOF model [117, 118]. The *2D* study focused on heavy oil IVF in cross-sections of horizontal wells [117]. The numerical model was validated against experimental data using *3D* simulations of VF in corefloods. It was found that the CFD model was capable of replicating IVF at mesoscale in *2D*, and in *3D* at core scale from the experimental validation. Moreover, it gave information about pressure and velocity profiles at different flow rates, along with predictions of breakthrough times. Lastly, the CFD model was shown to be able to calculate the critical velocity.

**Figure 20:**
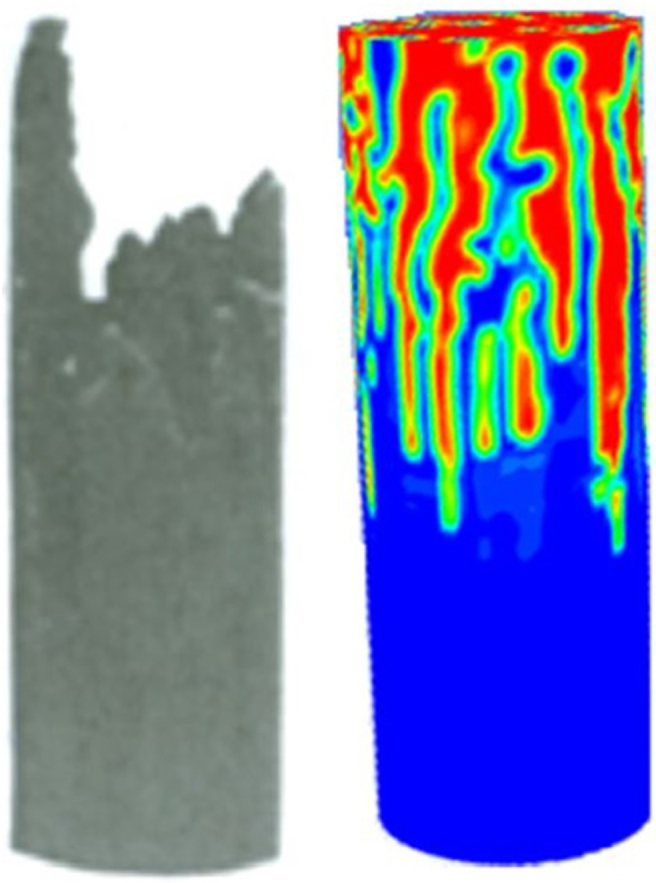
Modelling of VF at Core scale in *3D*, adapted from Wijeratne [117].

Wijeratne's second study was about the influence of Inflow Control Devices in horizontal wells for heavy oil recovery using *2D* simulations. Contrary to the first study, the transversal section of the reservoir and well were simulated based on the VOF method [118]. Additionally, the Inflow Control Devices were simulated using a pressure-flow function, avoiding the modeling of the specific device. It was determined that the CFD model could simulate IVF at mesoscale in horizontal wells with Inflow Control Devices. Moreover, the simulation provided information about the interface velocity and critical velocity, which explained the appearance of instabilities at very early stages. Interestingly, the simulation provided descriptive profiles of the phase distribution around the well and the Inflow Control Devices. It was demonstrated that water propagates (or finger growths) around the well, reducing oil production at breakthrough. Also, pressure drop near the wellbore or across the Inflow Control Devices is higher due to this phenomenon. Nevertheless, the author recognizes that the Inflow Control Devices effects study was limited or not enough due to the model's simplifications. It was suggested using a longer well with a higher amount of Inflow Control Devices, along with a *3D* model, to capture the effects of these devices adequately. Some of the results obtained by Wijeratne are presented in Figure 20.

An experimental and computational study of waterflooding in micromodels was conducted by Ahmadi [119] to determine the best location of injection-production wells depending on heterogeneous permeabilities, fractures, and injection flow rates. The experiments were carried on using glass micromodels of heterogeneous permeabilities and with fractured and non-fractured setups. Also, simulations on CFD were conducted to investigate further the impacts of using different flow rates and wettability conditions. The experiments and simulations were developed in 60 × 60mm domains. Also, two experimental setups with fractures were considered: i) the first one with an angular fracture pattern, and ii) a second setup with a parallel fracture pattern. It was found that the CFD model replicated the experimental results quantitatively and qualitatively describing VF. It was determined that injection wells should not be placed along the maximum pressure gradient line when fractures are present, leading to lower oil recoveries. Instead, it was suggested to find a location that achieves a proper angle with the fractures to mitigate the adverse effects. Also, it was determined that injection wells with low injection rates should be placed at the high permeability zones to achieve higher oil recoveries and delayed breakthrough times. Lastly, the results of different wettability conditions showed that water wet systems favor displacements increasing oil recovery.

## 7 Conclusions

This review has compiled some of the most significant literature about the study of VF. Decades of research have helped to elucidate the physical mechanisms that characterize this phenomenon. Miscible and immiscible displacements have been of primary interest due to their implications in subsurface processes, especially in oil recovery and *CO*_*2*_ sequestration. In this regard, it was evidenced that the research of this phenomenon has been mainly driven by the oil industry, which has a particular interest in comprehending VF due to its implications in oil production efficiency.

Several factors affect and trigger VF. For example, the wettability, porous physical properties, fluid properties, the miscibility between the phases, rheology, and fractality. Due to this complexity, there is no mathematical solution capable of describing multiphase flows in porous media. Several researchers have pointed the limitations of the Darcy Law and more complex and sophisticated mathematical models. Consequently, there has not been a solution capable of linking the microscopic influence of VF to its macroscopic consequences, such as conning and cresting.

Although VF is a well-characterized phenomenon, with extensive experimental study in *2D*, *3D* at the micro and macro scale, there has not been a consensus on which mathematical solution has been the most successful one in attempting to simulate this phenomenon. This article reviewed several solutions based on DLA methods, the Darcy Law, Hele-Shaw models, and conservation equations. Therefore, it dares to suggest that the most successful solutions have arisen from the conservation equations of fluid flow, especially the Navier-Stokes equations for immiscible displacements. In this regard, it suggests that future studies should focus on solutions based on these mathematical equations, for example, in solutions based on Lattice-Boltzmann modeling, or the Galerkin method, or other solutions based on the finite volume methods. Moreover, due to the high non-linearity of VF, more sophisticated equations of a higher order should be considered. In this regard, solutions based on the Burnett equations should be explored to model multiphase flow in porous media.

## Declarations

### Author contribution statement

All authors listed have significantly contributed to the development and the writing of this article.

### Funding statement

This research did not receive any specific grant from funding agencies in the public, commercial, or not-for-profit sectors.

### Data availability statement

No data was used for the research described in the article.

### Declaration of interests statement

The authors declare no conflict of interest.

### Additional information

No additional information is available for this paper.

## Footnotes

1 NR: Not Reported.

2 RTM: Refer to manuscript. Some data is presented in terms of fields, profiles or ratios.
